# Electronic Modulation via a Pd-CeO_2_ Heterointerface for Superior Alkaline Hydrogen Oxidation

**DOI:** 10.3390/molecules31081306

**Published:** 2026-04-17

**Authors:** Minhui Zhong, Qingzhen Xu, Wenhai Xu, Wei Zhang, Man Zhao, Yizhe Li, Wen Liu

**Affiliations:** 1 Department of Chemistry, Beijing University of Chemical Technology, Beijing 100029, China; 2024210893@buct.edu.cn (M.Z.); xuqingzhen1208@163.com (Q.X.); 2025400370@buct.edu.cn (W.X.); 2024400344@buct.edu.cn (W.Z.); 2 CAS Key Laboratory of Nanosystem and Hierarchical Fabrication, National Center for Nanoscience and Technology, Beijing 100190, China

**Keywords:** electrocatalysis, alkaline hydrogen oxidation, heterointerface, electronic modulation, Pd-based catalysts

## Abstract

The sluggish kinetics of the hydrogen oxidation reaction (HOR) in alkaline media remain a primary bottleneck for anion exchange membrane fuel cells (AEMFCs), necessitating catalysts that synergistically optimize the adsorption of hydrogen (*H) and hydroxide (*OH) intermediates. Herein, we construct a well-defined heterointerface between Pd clusters and CeO_2_ on nitrogen-doped carbon (Pd-CeO_2_/NC) to electronically engineer the active sites. Spectroscopic studies and theoretical calculations collectively reveal that CeO_2_ acts as an electron acceptor, drawing electrons from Pd via interfacial Pd-O-Ce bridges. This charge transfer induces a downshift of the Pd d-band center, which optimally tunes the adsorption strength of both *H and *OH at the interface, thereby breaking the scaling relationship that limits HOR activity. The resulting Pd-CeO_2_/NC catalyst achieves an exceptional exchange current density of 3.66 mA cm^−2^, surpassing that of commercial Pt/C by a factor of two and ranking among the best reported noble metal catalysts. Furthermore, it exhibits outstanding long-term stability and remarkable CO tolerance, retaining high activity in an atmosphere containing 1000 ppm CO. This work underscores the profound efficacy of metal–oxide heterointerface engineering in regulating electronic structures for multi-intermediate optimization, offering a viable design principle for advanced alkaline HOR electrocatalysts.

## 1. Introduction

The global transition toward a carbon-neutral energy system has intensified the focus on hydrogen as a clean and efficient secondary energy carrier [1,2,3]. In this context, anion exchange membrane fuel cells (AEMFCs) stand out as one of the most promising technologies for hydrogen utilization [4,5]. However, their widespread commercialization is severely hindered by the sluggish kinetics of the hydrogen oxidation reaction (HOR) in alkaline electrolytes, which is approximately two orders of magnitude slower than in acidic media [6,7]. This kinetic bottleneck is intrinsically linked to the suboptimal adsorption energetics of key reaction intermediates. According to the well-established bifunctional mechanism, the concurrent optimization of hydrogen (*H) and hydroxide (*OH) intermediate adsorption on the catalyst surface is crucial for efficient HOR/HER kinetics [8]. For instance, the decoration of Pt surfaces with Ni(OH)_2_ clusters, which provide active *OH sites, has been shown to significantly enhance HOR activity, underscoring the importance of balancing *H and *OH binding strengths [9].

While platinum-based catalysts currently demonstrate the highest activity for the alkaline HOR, their practical application is constrained by limited natural abundance, a high cost, and susceptibility to carbon monoxide (CO) poisoning [10,11,12]. Palladium (Pd) has emerged as a promising alternative due to its greater abundance, superior CO tolerance, and excellent stability in alkaline environments [13,14,15]. Nevertheless, the inherently strong hydrogen binding energy (HBE) on pure Pd surfaces often makes H* desorption the rate-determining step, occupying active sites and consequently impeding the overall HOR kinetics [16]. To address this limitation, heterointerface engineering has proven to be a powerful strategy for precisely tailoring the electronic structures of catalysts, thereby optimizing the adsorption behavior of intermediates and boosting intrinsic activity [17,18,19]. Introducing a second component to construct interfaces with Pd, such as in core–shell structures or metal–phosphide heterojunctions, can effectively modulate the HBE through interfacial charge rearrangement, leading to remarkable performance enhancements [15,20,21].

The judicious selection of this secondary component is critical. Among various candidates, cerium oxide (CeO_2_) is particularly attractive for constructing advanced heterointerfaces. Its unique properties, including tunable oxygen vacancy defects and a flexible Ce^3+^/Ce^4+^ redox couple, endow it with a remarkable oxygen storage capacity and a potent electron-regulating capability [22]. We posit that by forming an intimate interface with Pd, CeO_2_ can serve as an effective electronic modulator. The multivalent nature of Ce may facilitate spontaneous charge transfer across the Pd-CeO_2_ interface, fine-tuning the electronic structure of Pd and optimizing its HBE. Concurrently, CeO_2_ itself exhibits a strong affinity for adsorbing and activating OH^−^ species [23], which could promote the OH^−^-involving step in the HOR pathway. This synergy offers a promising route to simultaneously regulate both *H and *OH intermediates, potentially lowering the overall reaction barrier.

In this work, we designed and synthesized a heterostructure catalyst comprising Pd clusters interfaced with CeO_2_ nanoparticles, supported on nitrogen-doped carbon (Pd–CeO_2_/NC). We hypothesized that the constructed Pd-CeO_2_ heterointerface would induce electron transfer from Pd to CeO_2_ via newly formed Pd-O-Ce bridges, leading to a downshift of the Pd d-band center. This electronic modulation was anticipated to synergistically weaken the HBE on Pd sites while strengthening the hydroxide binding energy (OHBE) on the interface, thereby breaking the scaling relationship that limits HOR activity. As anticipated, the Pd-CeO_2_/NC catalyst demonstrated exceptional alkaline HOR performance, surpassing commercial Pt/C in activity, durability, and CO tolerance. Combined spectroscopic analysis and theoretical calculations confirm that the interfacial charge redistribution creates optimal active sites for stabilizing both *H and *OH, substantially reducing the reaction energy barrier. This study elucidates the fundamental benefits of metal oxide heterointerface engineering and provides a viable design principle for developing high-performance, durable HOR electrocatalysts.

## 2. Results and Discussion

### 2.1. Catalyst Synthesis and Characterization

**Figure 1:**
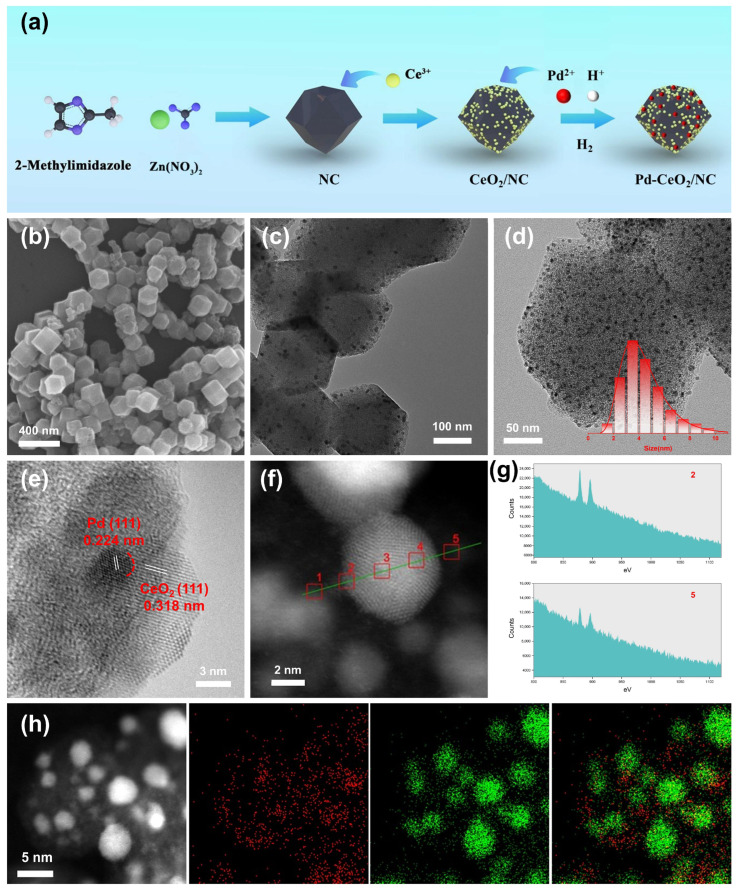
Synthesis and morphological characterization of the Pd–CeO_2_/NC catalyst. (**a**) Schematic illustration of the synthesis procedure for Pd-CeO_2_/NC; (**b**) SEM image of the nitrogen-doped carbon (NC) support, displaying its rhombic dodecahedron morphology; (**c**) TEM image of CeO_2_/NC; (**d**) TEM image of Pd-CeO_2_/NC, with the inset showing the corresponding particle size distribution histogram; (**e**) HRTEM image of a Pd-CeO_2_/NC nanoparticle, where the lattice fringes with spacings of 0.224 nm and 0.318 nm correspond to the (111) planes of Pd and CeO_2_, respectively, confirming the formation of a heterointerface; (**f**) HAADF-STEM image of a selected Pd–CeO_2_ cluster and the path for the EELS line scan. The red numbers and boxes in the figure represent different regional electron energy loss spectroscopy (EELS) line scans; (**g**) corresponding EELS line scan profile for the Ce signal across the cluster in (**f**), demonstrating the concentration of Ce at the interface; (**h**) HAADF-STEM image and the corresponding EDS elemental maps for Pd and Ce, illustrating the spatial distribution where CeO_2_ surrounds the Pd nanoparticles.

**Figure 2:**
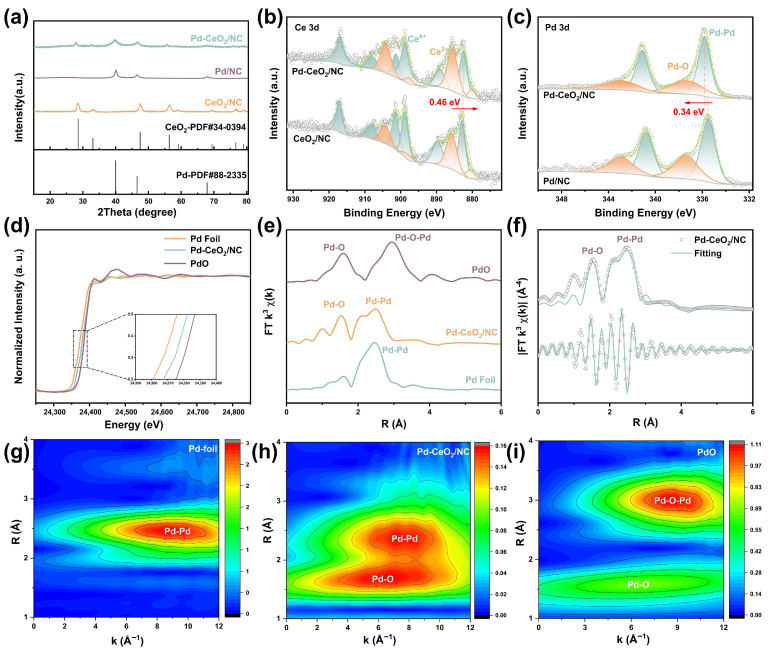
Structural and electronic characterizations of the Pd–CeO_2_ interface. (**a**) XRD patterns of Pd-CeO_2_/NC, CeO_2_/NC, and Pd/NC, confirming the co-existence of crystalline Pd and CeO_2_ phases. (**b**) High-resolution Ce 3d XPS spectra of Pd-CeO_2_/NC and CeO_2_/NC. The negative shift and altered Ce^3+^/Ce^4+^ ratio in Pd–CeO_2_/NC indicate electron transfer at the interface. (**c**) High-resolution Pd 3d XPS spectra of Pd-CeO_2_/NC and Pd/NC. The positive binding energy shift in the Pd peaks in Pd–CeO_2_/NC confirms electron donation from Pd to CeO_2_. (**d**) Pd K-edge XANES spectra. (**e**) k^3^-weighted FT-EXAFS spectra for Pd–CeO_2_/NC and reference samples. (**f**) EXAFS fitting for Pd–CeO_2_/NC in R-space, revealing Pd–O and Pd–Pd coordination shells. Corresponding wavelet transform (WT) contour plots of the EXAFS signals for (**g**) Pd foil; (**h**) Pd–CeO_2_/NC, and (**i**) PdO. The WT plots provide enhanced resolution in both R- and k-space, clearly distinguishing the Pd-O and Pd-Pd scattering paths.

A schematic illustration of the synthesis process for the Pd–CeO_2_ heterostructure on nitrogen-doped carbon (Pd-CeO_2_/NC) is presented in Figure 1a. The synthesis commenced with the preparation of a porous nitrogen-doped carbon (NC) support. Using ZIF-8 as a self-sacrificial template, a pyrolysis strategy yielded NCs with a well-defined rhombic dodecahedron morphology and an approximate diameter of 200 nm, as confirmed by scanning electron microscopy (SEM) (Figure 1b and Figure S1). Subsequently, CeO_2_ was anchored onto the NC support. Briefly, the as-prepared NC was uniformly dispersed and mixed with a cerium acetylacetonate precursor. After vacuum drying, the obtained precursor was thermally treated under an argon atmosphere (heated at 120 °C for 3 h, followed by pyrolysis at 900 °C for 2 h) to form CeO_2_/NC. The successful formation of CeO_2_ was verified by X-ray diffraction (XRD), with diffraction patterns matching the standard CeO_2_ phase (PDF#34-0394) (Figure 2a and Figure S3). Transmission electron microscopy (TEM) images confirmed the dispersion of CeO_2_ nanoparticles on the NC framework (Figure 1c and Figure S2). The final step involved the introduction of Pd to construct the heterointerface. A palladium precursor was introduced to the CeO_2_/NC dispersion. Notably, to regulate the interfacial structure, we employed a strongly acidic palladium nitrate solution as the Pd source. Following adsorption and drying, the material was reduced under a H_2_ atmosphere at an elevated temperature to obtain the final Pd-CeO_2_/NC catalyst (see Supporting Information for details). For comparison, a control sample labeled Pd-CeO_2_ (Big)/NC was synthesized using a neutral palladium chloride solution. The morphology and structure of the catalysts were thoroughly characterized. TEM analysis of Pd-CeO_2_/NC (Figure 1d) revealed nanoparticles with an average size of 4.0 nm uniformly dispersed on the NC support. Remarkably, the Pd particles in Pd-CeO_2_/NC were significantly smaller than those in the Pd/NC control sample (Figure S4, Supporting Information), suggesting a confinement effect imposed by the pre-formed CeO_2_, which likely inhibits Pd aggregation during reduction. The XRD pattern of Pd-CeO_2_/NC (Figure 2a) exhibited characteristic peaks for both metallic Pd and CeO_2_, confirming the successful co-existence of both crystalline phases. We further optimized the catalyst composition by varying the CeO_2_ loading. TEM observations (Figure S5, Supporting Information) indicated that an addition of 15 mg cerium acetylacetonate yielded CeO_2_ nanoparticles that were small and uniformly distributed on NCs. Deviations from this optimal amount resulted in either sparse coverage (low Ce) or severe agglomeration (excessive Ce), both detrimental to active site exposure, a trend consistent with subsequent electrochemical performance. A pivotal finding was the profound impact of the Pd precursor’s chemical state. Comparative analysis showed that using the strongly acidic Pd(NO_3_)_2_ solution, as opposed to neutral PdCl_2_, yielded Pd-CeO_2_/NC with notably smaller and more uniformly distributed Pd and CeO_2_ nanoparticles (Figures S6 and S7). We attribute this to an in situ acid etching effect during the Pd^2+^ incorporation step. The introduced acidic environment likely refines the CeO_2_ support surface and modulates the Pd nucleation process, ultimately fostering a more intimate and finely structured Pd-CeO_2_ interface, which is crucial for interfacial catalysis.

The formation of an intimate heterointerface was observed through high-resolution transmission electron microscopy (HRTEM). As shown in Figure 1e, the HRTEM image of a representative nanoparticle reveals two distinct sets of lattice fringes. The measured interplanar spacings of 0.318 nm and 0.224 nm correspond to the (111) planes of CeO_2_ and metallic Pd, respectively. The clear observation of these lattice fringes in direct contact provides definitive visual proof of a Pd-CeO_2_ heterojunction. To further probe the local chemical composition across this interface, electron energy loss spectroscopy (EELS) line scans were performed (Figure 1f). The corresponding profile of the Ce signal (Figure 1g) is highly revealing: a significant Ce signal is detected only at the boundary regions between the Pd core and the surrounding material (positions 2 and 5 in Figure 1f), while it drops to background levels within the interior of the nanoparticle. This result unambiguously indicates that CeO_2_ is distributed around the Pd nanoparticles, maintaining close contact and forming a Pd-CeO_2_ heterointerface (Figure S8). The spatial distribution of elements was further corroborated by high-angle annular dark-field scanning transmission electron microscopy (HAADF-STEM) coupled with energy-dispersive X-ray spectroscopy (EDS) mapping. As presented in Figure 1h and Figure S9, the elemental maps for Pd and Ce show a clear correlation. The Pd signal is concentrated in discrete nanoparticle regions, whereas the Ce signal forms a complementary shell that closely encases the Pd, creating a distinct spatial heterointerface. This configuration, with Pd nanoparticles ensheathed by CeO_2_, is fully consistent with the HRTEM and EELS findings.

To gain insights into the electronic structure and chemical states induced by the heterointerface, X-ray photoelectron spectroscopy (XPS) analysis was conducted on Pd-CeO_2_/NC and the reference samples (CeO_2_/NC and Pd/NC) (Figures S10 and S11). The C 1s spectrum of Pd-CeO_2_/NC could be deconvoluted into characteristic peaks corresponding to C-C, C-O/C-N, and C=O bonds, confirming the successful co-doping of nitrogen and oxygen into the carbon matrix, which is known to enhance the electrical conductivity of the support. In Figure 2b, the high-resolution Ce 3d XPS spectrum was fitted with five spin–orbit doublets, primarily corresponding to Ce^3+^ and Ce^4+^ states. Compared to CeO_2_/NC, the peaks for Pd-CeO_2_/NC shifted to a lower binding energy by 0.46 eV. Concurrently, a higher Ce^3+^/Ce^4+^ ratio was observed, suggesting a greater concentration of defects and oxygen vacancies in CeO_2_. This is further corroborated by the O 1s spectrum of Pd-CeO_2_/NC (Figure S12), where the ratio of oxygen vacancy-associated species (Vacancy-O) to lattice oxygen (Lattice-O) increased significantly from 1.67 in CeO_2_/NC to 3.45 in Pd-CeO_2_/NC. The elevated Ce_3+_ content and oxygen vacancy concentration are strong indicators of enhanced reducibility and electron-accepting capability in the interfacial CeO_2_.

The mixed valence states of Ce (Ce^3+^/Ce^4+^) enable fine-tuning of the electronic structure of Pd via spontaneous charge transfer across the intimate heterointerface. This modulation is anticipated to optimize the adsorption energy of key reaction intermediates and lower the reaction energy barrier. The Pd 3d spectrum of Pd-CeO_2_/NC (Figure 2c) was deconvoluted into two spin–orbit doublets, corresponding predominantly to metallic Pd0 with a minor contribution from Pd^2+^. The binding energies for the Pd 3d_5/2_ and Pd 3d_3/2_ peaks of Pd^0^ were measured at 335.48 eV and 337.38 eV, respectively. These peaks exhibit a distinct positive shift of 0.34 eV compared to those in Pd/NC, demonstrating electron transfer from Pd to Ce at the interface. This systematic shift to higher binding energy provides direct evidence for electron transfer from Pd to CeO_2_ across the heterointerface, rendering the Pd sites electron deficient.

X-ray absorption spectroscopy (XAS) was subsequently employed to gain deeper insights into the electronic structure and coordination environment of Pd in Pd-CeO_2_/NC. The Pd K-edge X-ray absorption near-edge structure (XANES) spectrum of Pd-CeO_2_/NC is presented in Figure 2d. The absorption edge energy of Pd-CeO_2_/NC lies between that of metallic Pd foil and PdO reference (inset of Figure 2d), indicating an average Pd oxidation state higher than Pd^0^ but lower than Pd^2+^. This intermediate valence state is a direct signature of the electronic interaction at the Pd-CeO_2_ interface, resulting from partial charge transfer and the formation of Pd-O-Ce bonds.

The local coordination configuration was further probed by extended X-ray absorption fine structure (EXAFS) spectroscopy. The k^3^-weighted Fourier transform spectra (FT-EXAFS) are shown in Figure 2e,f, with detailed fitting parameters summarized in Table S1. The quality of the k-space data directly influences the reliability of the fitted structure, and the intense Pd K-edge signals for both Pd-CeO_2_/NC and Pd foil (Figures S13 and S14) validate the accuracy of the fitting results. The spectrum of Pd-CeO_2_/NC displays two prominent peaks. The first peak, located at approximately 1.53 Å (not corrected for phase shift), is attributed to Pd-O coordination. The second peak, near 2.47 Å, corresponds to Pd–Pd bonds. This confirms the coexistence of oxidized Pd species (bonded to oxygen) and metallic Pd clusters in the catalyst. Notably, the fitted Pd-O bond length in Pd-CeO_2_/NC is shorter than that in a standard PdO reference. This bond contraction strongly suggests a specific and strong bonding interaction between Pd atoms and the oxygen lattice of the adjacent CeO_2_ at the heterointerface, consistent with the formation of interfacial Pd-O-Ce linkages.

Furthermore, wavelet transform (WT), known for its high resolution in both R and k-spaces, was applied to the k^3^-weighted EXAFS signals at the Pd K-edge for Pd-CeO_2_/NC. The WT-EXAFS contour plots (Figure 2g–i) clearly resolve the intensity maxima corresponding to the Pd-O and Pd–Pd coordination shells. The clear separation and intensity of these features in the WT domain provide robust, high-resolution confirmation of the dual coordination environment around Pd atoms, as deduced from the conventional EXAFS fitting.

Collectively, the combined XPS and XAS results deliver a consistent and powerful narrative of interfacial electronic engineering. XPS revealed electron transfer from Pd to CeO_2_, evidenced by a positive shift in Pd binding energy and an increased Ce^3+^/Ce^4+^ ratio. XAS provides atomic-level structural validation: the intermediate oxidation state from XANES and the shortened Pd-O bond from EXAFS directly evidences the covalent interaction at the Pd-CeO_2_ interface. This charge redistribution, mediated by Pd-O-Ce bridges, dynamically modulates the electronic structure of Pd, most notably by downshifting its d-band center. As established in catalysis theory, such a downshift is a key descriptor for optimizing the adsorption strength of reaction intermediates. Therefore, this electronically modulated interface is identified as the fundamental reason for the subsequent enhancement in alkaline HOR kinetics, as it promises to optimally balance the binding energies of both *H and *OH species [24].

### 2.2. Electrocatalytic Performance for Alkaline HOR

To evaluate the impact of the interfacial interaction between Pd and CeO_2_ on the catalytic activity, the HOR electrocatalytic performance of Pd-CeO_2_/NC, CeO_2_/NC, Pd/NC, NC, and commercial 20 wt.% Pt/C catalysts was assessed using a rotating disk electrode (RDE) in H_2_-saturated 0.1 M KOH electrolyte. Prior to the main performance comparison, key synthesis parameters were optimized. The relationship between the Pd/Ce molar ratio and HOR activity was investigated, identifying an optimal ratio of approximately 2.6:1 (Figure S15), which aligns well with the mass ratio of 2.8:1 determined by inductively coupled plasma optical emission spectrometry (ICP-OES, Table S2). Furthermore, the strategic use of a strong acidic Pd precursor, as discussed in Section 2.1, was confirmed to be crucial. This “acid etching” treatment yielded finer and more uniformly dispersed Pd and CeO_2_ nanoparticles (Figures S6, S7, S10 and S16), thereby maximizing the exposure of active interfacial sites and contributing to enhanced activity.

**Figure 3:**
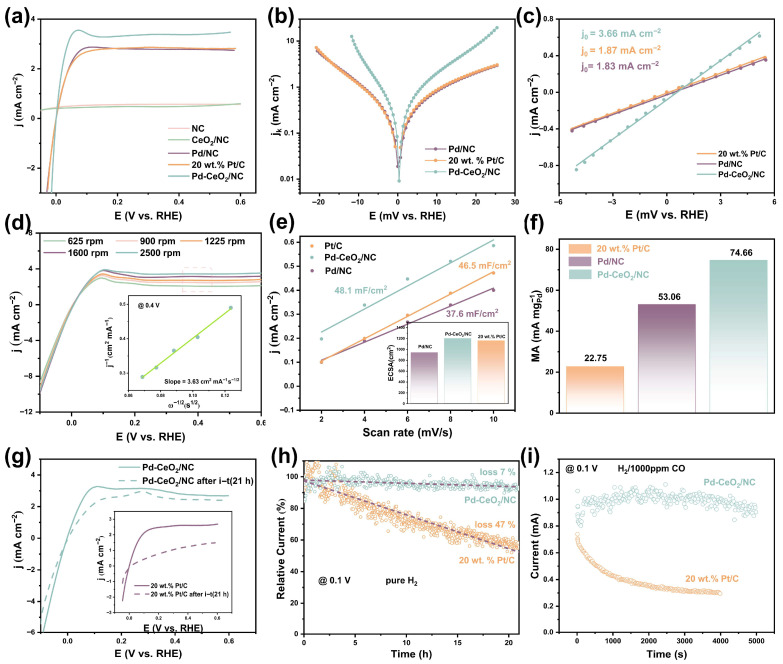
Electrochemical HOR activity and stability of the catalyst. (**a**) HOR polarization curves (after 90% iR-compensation) measured in H_2_-saturated 0.1 M KOH solution at the rotation rate of 1600 rpm and a scan rate of 5 mV s^−1^. (**b**) Corresponding Tafel plots of the kinetic current density (j_k_) derived from (**a**) using the Koutecký–Levich equation. (**c**) Micropolarization region (−5 to 5 mV vs. RHE) of Pd-CeO_2_/NC, Pd/NC, and 20 wt.% Pt/C. The dashed lines are linear fits used to determine the exchange current density (j_0_). (**d**) HOR polarization curves of Pd-CeO_2_/NC at different rotation rates. The inset shows the corresponding Koutecký–Levich plot at an overpotential of 0.4 V, with the slope indicating the number of electrons transferred. The dashed box represents the current density at around 0.4 V. (**e**) Double-layer capacitance (C_dl_) measurements used to estimate the electrochemical active surface area (ECSA). The inset compares the calculated ECSA values for the three catalysts. (**f**) Comparison of the mass activity (normalized to the mass of platinum-group metal, PGM) at an overpotential of 50 mV. (**g**) Comparison of HOR polarization curves for Pd-CeO_2_/NC and Pt/C before (solid lines) and after (dashed lines) a 21 h accelerated durability test (ADT). (**h**,**i**) Chronoamperometry (i-t) tests performed at 0.1 V vs. RHE and 1600 rpm: (**h**) in pure H_2_-saturated 0.1 M KOH, and (**i**) in a H_2_/1000 ppm CO mixture-saturated 0.1 M KOH.

The HOR polarization curves are presented in Figure 3a and Figure S17. Pd-CeO_2_/NC demonstrates superior performance, exhibiting the most rapid current increase and achieving the highest limiting diffusion current density (3.35 mA cm^−2^). This value substantially exceeds those of benchmark commercial Pt/C (2.86 mA cm^−2^) and the control catalyst Pd/NC (2.79 mA cm^−2^). Notably, Pd-CeO_2_/NC requires an overpotential of only 45 mV to reach the limiting diffusion current, which is less than half of that required for Pt/C (100 mV), highlighting its more favorable reaction kinetics. In contrast, CeO_2_/NC showed negligible HOR activity, confirming that Pd is the essential active component, while CeO_2_ plays a critical promotional role. The significant enhancement in both kinetics and limiting current for Pd-CeO_2_/NC over Pd/NC is a direct consequence of the constructed heterointerface, which optimizes the adsorption energetics of key intermediates (*H and *OH).

To quantitatively deconvolute the kinetic contribution, the polarization data were analyzed using the Koutecký–Levich equation. The derived kinetic current densities are plotted in the form of Tafel plots in Figure 3b and Figure S18. Pd-CeO_2_/NC exhibits the smallest Tafel slope, confirming it possesses the most rapid HOR kinetics among all tested catalysts. The intrinsic activity was further quantified by the exchange current density (j_0_), obtained from linear fitting in the micro-polarization region (Figure 3c). Pd-CeO_2_/NC delivers an exceptional j0 of 3.66 mA cm^−2^, which is double that of both Pd/NC (1.83 mA cm^−2^) and commercial Pt/C (1.87 mA cm^−2^). This value ranks among the highest reported for platinum-group metal-based HOR electrocatalysts (see comparative Table S3), underscoring the efficacy of the interface engineering strategy.

The mass-transfer characteristics and reaction pathway were validated by collecting polarization curves at various rotation rates (Figure 3d and Figure S19). The corresponding Koutecký–Levich (K-L) plot at 0.4 V overpotential yields a slope value of 3.63 cm^2^ mA^−1^ rpm^−1/2^, which is close to the theoretical value of 4.87 cm^2^ mA^−1^ rpm^−1/2^ for the two-electron hydrogen oxidation reaction (HOR), confirming the measured anodic current is predominantly from H_2_ oxidation [25]. The electrochemical active surface area (ECSA), estimated from double-layer capacitance measurements (Figure 3e and Figure S20), was 1102.5 cm^2^ for Pd-CeO_2_/NC, larger than those of Pd/NC and Pt/C. This increased ECSA contributes to, but cannot solely account for, the dramatic activity enhancement. To provide a techno-economically relevant metric, the mass activity (MA) normalized to the platinum-group metal (PGM) mass was calculated (Figure 3f). Pd-CeO_2_/NC achieves a superior MA of 74.66 mA mg_PGM_^−1^, which is 3.28 and 1.41 times higher than that of Pt/C (22.76 mA mg_PGM_^−1^) and Pd/NC (53.07 mA mg_PGM_^−1^), respectively. This demonstrates the high utilization efficiency of precious metals enabled by the interfacial design.

For practical AEMFC applications, stability and impurity tolerance are as critical as initial activity. The durability of Pd-CeO_2_/NC was assessed via accelerated degradation tests (ADT) involving 21 h of continuous potential cycling between −0.1 and 0.6 V at a scan rate of 50 mV s^−1^. As shown in Figure 3g, Pd-CeO_2_/NC exhibits minimal current decay, whereas Pt/C suffers a degradation exceeding 50%. This superior stability was further confirmed by a 21 h chronoamperometry (i-t) test at a constant potential (Figure 3h), with Pd-CeO_2_/NC retaining ~93% of its initial current compared to only ~53% for Pt/C. We further evaluated the structural features of these catalysts after durability tests to discern the alterations that had occurred. The XRD pattern of the Pd-CeO_2_/NC remains the same without obvious changes after testing compared with fresh Pd-CeO_2_/NC, indicating the structural stability of the Pd-CeO_2_/NC during testing (Figure S21). Meanwhile, cyclic voltammetry tests were conducted on the samples before and after durability tests (Figure S22). The results show that the hydrogen adsorption/desorption peaks and the palladium redox peaks remain clearly observable in the cyclic voltammetry curves after durability tests, indirectly confirming the structural stability of the Pd-CeO_2_/NC catalyst. Furthermore, resistance to carbon monoxide (CO) poisoning is a critical challenge for fuel cells operating with reformate hydrogen. In an atmosphere containing 1000 ppm CO/H_2_, the HOR current on Pd-CeO_2_/NC showed negligible decay over 5000 s, while Pt/C was virtually deactivated within 4000 s (Figure 3i).

The electrochemical data collectively establish Pd-CeO_2_/NC as a superior alkaline HOR catalyst. It simultaneously achieves high intrinsic activity (j_0_), excellent mass-specific activity, outstanding long-term stability, and remarkable CO tolerance, surpassing the benchmark Pt/C on all fronts. This comprehensive enhancement unequivocally stems from the synergistic interaction at the Pd–CeO_2_ heterointerface. The interface not only provides more active sites (higher ECSA) but, more importantly, modulates the electronic structure of Pd to optimize intermediate adsorption, thereby boosting the intrinsic kinetics. The stable interfacial structure also appears to anchor the Pd species and mitigate degradation pathways, while the modified electronic environment weakens CO binding, explaining the enhanced durability and poisoning resistance.

### 2.3. Mechanism Investigation

**Figure 4:**
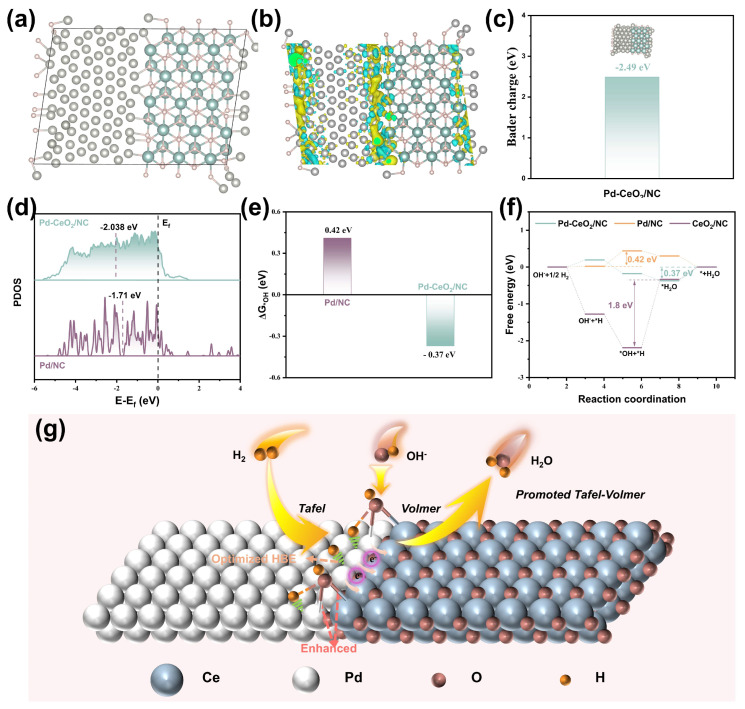
DFT-based mechanistic investigation of the alkaline HOR on the Pd-CeO_2_ heterointerface. (**a**) Top view atomic model of the Pd-CeO_2_ heterostructure. The gray, cyan, and pink spheres represent Pd, Ce, and O atoms, respectively. (**b**) Differential charge density distribution at the Pd-CeO_2_ interface. The yellow and olive surfaces denote regions of electron accumulation and depletion, respectively, indicating electron transfer from Pd to CeO_2_. (**c**) Corresponding Bader charge analysis quantifying the net electron transfer (approximately 2.49 |e|) from the Pd cluster to CeO_2_. (**d**) Projected density of states (PDOS) for the Pd 3d orbitals in Pd-CeO_2_/NC and Pd/NC. The vertical dashed lines mark the calculated d-band centers (ε_d_), showing a downshift for Pd at the heterointerface. (**e**) Calculated adsorption free energy (ΔG_*OH_) for the hydroxide intermediate (OH) on Pd-CeO_2_/NC and Pd/NC. (**f**) Computed Gibbs free energy diagram for the alkaline HOR pathway on Pd-CeO_2_/NC, CeO_2_/NC, and Pd/NC, based on the Tafel–Volmer mechanism. The rate-determining steps (RDS) are indicated. (**g**) Schematic illustration of the proposed HOR mechanism at the Pd-CeO_2_ heterointerface in alkaline media, highlighting the synergistic roles of Pd (for H_2_ dissociation/H* adsorption) and CeO_2_ (for OH^−^ adsorption/activation).

To elucidate the fundamental origin of the enhanced HOR performance at the electronic level, density functional theory (DFT) calculations were performed. The structural models were constructed based on experimental characterizations, encompassing pristine Pd(111), CeO_2_, and a heterointerface model between Pd nanoclusters (NCs) and CeO_2_ (Figure 4a and Figure S23). In the interface model, Pd and CeO_2_ are connected through Pd-O-Ce covalent bridges, which serve as the channels for electronic communication.

The charge density difference plot (Figure 4b) vividly illustrates a net electron depletion around Pd atoms and accumulation around the interfacial Ce atoms, confirming electron transfer from Pd to CeO_2_. Bader charge analysis quantifies this transfer, revealing that approximately 2.49 electrons are donated from the Pd cluster to the adjacent CeO_2_ at the interface (Figure 4c). This result is in excellent agreement with the experimental XPS and XAS findings, providing theoretical validation for the interfacial charge redistribution. The generation of electron-deficient Pd sites is anticipated to modulate its electronic structure, particularly the energy of the d-band center. The projected density of states (PDOS) analysis confirms this modulation: the calculated d-band center (ε_d_) for Pd in the heterointerface is downshifted to −2.04 eV, compared to −1.71 eV for isolated Pd NCs (Figure 4d). This downshift is a critical electronic descriptor that typically correlates with a weakened adsorption strength for hydrogen (*H), thereby optimizing the hydrogen binding energy (HBE). Concurrently, the Pd 3d orbitals in the heterostructure exhibit greater delocalization and a higher density of occupied states near the Fermi level (Figure S24), indicating enhanced metallicity and electron conductivity, which benefits charge transfer during electrocatalysis [26].

The HOR activity of Pd-CeO_2_/NC is governed by the adsorption energies of key reaction intermediates, *H and *OH [27,28]. The optimal adsorption configurations on different models are shown in Figures S25–S27. A key finding is that on the Pd-CeO_2_ interface, H preferentially adsorbs at a three-fold hollow site composed of Pd atoms, while OH stabilizes at a unique bridge site involving both Pd and Ce atoms. This spatial separation allows for the concurrent and optimized stabilization of both intermediates. Quantitative analysis of the adsorption free energies reveals that the heterointerface engineering achieves a dual optimization (Figure 4e and Figure S28): it weakens the H adsorption (ΔG_*H_) on Pd sites compared to pure Pd, while significantly strengthening the OH adsorption (ΔG_*OH_ = −0.37 eV) relative to pure Pd NCs (ΔG_*OH_ = 0.42 eV). This synergistic modulation breaks the scaling relationship between H and OH adsorption, creating a near-optimal balance that is crucial for accelerating the alkaline HOR kinetics. To map the complete reaction pathway, the Gibbs free energy profile was constructed based on the prevailing Tafel–Volmer mechanism (Figure 4f). For isolated Pd NCs, the adsorption of OH^−^ is highly endergonic (0.42 eV), constituting the rate-determining step (RDS). In stark contrast, for the Pd-CeO_2_ interface, the favorable OH^−^ adsorption lowers this barrier, shifting the RDS to the desorption of H_2_O, with a substantially reduced overall barrier of 0.37 eV. This 0.05 eV reduction in the activation barrier translates to a significant kinetic enhancement, consistent with the experimental exchange current density measurements.

A comprehensive reaction mechanism is proposed in Figure 4g. The interfacial Pd-O-Ce bridges facilitate electron transfer from Pd to CeO_2_, rendering Pd electron-deficient and downshifting its d-band center. This electronic state optimally tunes the H* binding strength. Concurrently, the Lewis acidic Pd-Ce pairs at the interface act as highly active sites for the adsorption and activation of OH^−^ species, increasing their local surface concentration. The proximate, optimally adsorbed *H and *OH intermediates then readily couple to form H_2_O, which subsequently desorbs. In essence, the Pd-CeO_2_ heterointerface functions as a bifunctional platform that synergistically promotes both the Tafel (H_2_ dissociation) and Volmer (*H + OH^−^ → H_2_O) steps, thereby dramatically enhancing the overall HOR activity, stability, and CO tolerance.

## 3. Experimental Section

### 3.1. Chemicals and Materials

2-methylimidazole (C_4_H_6_N_2_), zinc nitrate hexahydrate (Zn(NO_3_)_2_·6H_2_O), cerium acetylacetonate (Ce(acac)_3_), palladium nitrate (Pd(NO_3_)_2_), palladium chloride (PdCl_2_), potassium hydroxide (KOH), anhydrous methanol, anhydrous ethanol, nitric acid, Nafion solution (~5% in a mixture of lower aliphatic alcohols and water), commercial 20 wt.% Pt/C catalyst were purchased from Johnson Matthey Corp (Suzhou, China), the deionized water (18.25 MΩ cm^−1^). All the chemicals were analytical grade and used without further purification.

### 3.2. Synthesis of NC

First, weigh 2.94 g of Zn(NO_3_)_2_·6H_2_O and 3.24 g of 2-methylimidazole and dissolve them in 150 mL of methanol. Ultrasonicate for 30 min to ensure complete dissolution. Place the beaker containing this solution on a magnetic stirrer and stir at room temperature for 12 h to complete the reaction. Centrifuge the solution and wash with methanol. Finally, transfer the centrifuge-separated sample to a freeze-drying apparatus and dry overnight to obtain ZIF-8. Place the ZIF-8 prepared in the previous step into a ceramic boat. Insert the ceramic boat into a quartz tube, which is then placed in a tube furnace. First, purge with Ar for 30 min to remove all air. Subsequently, initiate the programmed temperature ramping. Within the Ar atmosphere, increase the temperature at a rate of 5 °C/min to 900 °C and maintain at this temperature for 2 h. Upon completion of calcination, allow the sample to cool to room temperature before removal, yielding NC.

### 3.3. Synthesis of CeO_2_/NC

First, weigh 40 mg of NC and 15 mg of cerium acetylacetonate and disperse them in 15 mL of a water-ethanol mixture (C_2_H_5_OH:H_2_O = 1:2). Ultrasonicate for 30 min to achieve uniform dispersion. Transfer the dispersion to a round-bottom flask for rotary evaporation. After evaporation, dry the residue overnight in a vacuum drying oven. Grind the dried sample into powder, place it in a porcelain boat, and insert the boat into a quartz tube. Position the quartz tube in a tube furnace. First, purge with Ar for 30 min to evacuate air, then initiate the programmed temperature ramp. Under an Ar atmosphere, the temperature was first raised at 2 °C/min to 120 °C and held for 3 h. It was then raised at 5 °C/min to 900 °C and held for 2 h. After heating completion, the sample was cooled to room temperature to obtain CeO_2_/NC.

### 3.4. Synthesis of Pd-CeO_2_/NC

First, weigh 40 mg of CeO_2_/NC and disperse it in 30 mL of ethanol. Then add a strong acid-dissolved palladium nitrate solution (0.09 mM) and ultrasonicate for 40 min to achieve uniform dispersion. Transfer the mixture to a magnetic stirrer and stir for 12 h to complete the reaction. Subsequently, transfer the mixture to a round-bottom flask for rotary evaporation. After rotary evaporation, dry the mixture overnight in a vacuum drying oven. Grind the dried sample into powder and place it in a porcelain boat. Insert the porcelain boat into a quartz tube, then place the quartz tube into a tube furnace. Prior to programmed temperature ramping, purge with argon for 30 min to evacuate residual air. Heat at 5 °C/min to 400 °C under a 5% H_2_ atmosphere, then hold at 400 °C for 2 h to complete calcination. After cooling to room temperature, remove the sample to obtain Pd-CeO_2_/NC.

### 3.5. Synthesis of Pd-CeO_2_(Big)/NC

The synthesis procedure is identical to that of Pd-CeO_2_/NC, with the sole difference being the use of a neutral palladium chloride solution (PdCl_2_) as the Pd precursor.

### 3.6. Synthesis of Other Samples

The synthesis procedure is identical to that of Pd-CeO_2_/NC, with the only difference being the varying amounts of Pd and Ce added.

### 3.7. Material Characterization

The morphologies of the materials were recorded by using scanning electron microscope (SEM, Zeiss Supra55, accelerating voltage = 20 kV, Carl Zeiss AG, Oberkochen, Germany), transmission electron microscope (TEM, Hitachi 7700, accelerating voltage = 60 KV, Hitachi Group, Shanghai, China), high-resolution transmission electron microscopy (HRTEM, JEOL JEM-2100, accelerating voltage = 200 kV, JEOL Ltd., Tokyo, Japan) and aberration-corrected high-angle annular darkfield scanning transmission electron microscope (HAADF-STEM, JEOL JEM-ARM200F, operated at 200 kV, JEOL Ltd., Tokyo, Japan). The chemical composition of materials was obtained from ICP-OES measurement (Agilent 720ES, Agilent Technologies, Santa Clara, CA, USA). The crystalline structures of all the samples were identified by using Shimadzu XRD-6000 diffractometer (Cu Kα source, λ = 1.5418 Å, Shimadzu Corporation, Kyoto, Japan). X-ray photoelectron spectroscopy (XPS) was carried out on Thermo Electron ESCALAB 250 (Thermo Fisher Scientific, Waltham, MA, USA). The XAS at the Pd K-edge was recorded at BL20U station in Shanghai Synchrotron Radiation Facility.

### 3.8. Electrochemical Measurements

Electrochemical measurements were performed in a standard three-electrode system at CHI 760E electrochemical station (Chenhua, Shanghai, China). To prepare the working electrode, 5 mg of catalyst, 490 μL of isopropanol and 20 μL of 5 wt.% Nafion solution were mixed and sonicated for 30 min to prepare catalyst ink. Then, 10 μL of the catalyst ink was coated onto a glassy carbon electrode (GCE, diameter 5 mm, specific surface area 0.196 cm^2^) mounted on a rotating disk electrode (diameter 5 mm). Graphite rods and saturated calomel electrodes (SCE) serve as counter electrodes and reference electrodes, respectively. The conversion formula of the potential of the reference electrode to RHE is:

(1) $$E=E\left(SCE\right)+0.059\;*\;pH+0.241$$

For HOR test, linear sweep voltammetry (LSV) was performed in H_2_-saturated 0.1 M KOH solution at a scan rate of 5.0 mV s^−1^ and rotation speed of 1600 rpm with 90% iR compensation. The accelerated durability tests were carried out in 0.1M KOH electrolyte between −0.1 and 0.6 V at a scan rate of 50 mV s^−1^ for 21 h. The chronoamperometry measurement (i–t) was conducted at 0.1 V in H_2_-saturated 0.1 M KOH at 1600 rpm. The kinetic current density (j_k_) was extracted from Koutecký–Levich equation:

(2) $$\frac{1}{j}=\frac{1}{j_{d}}+\frac{1}{j_{k}}$$

where j is measured current and j_d_ is diffusion-limited current, which can be collected by the Levich equation:

(3) $$j_{d}=0.62nFD^{2/3}v^{-1/6}C_{0}\omega^{1/2}=BC_{0}\omega^{1/2}$$

where n, F, D, v, C_0_, and ω are the number of electrons transferred (2), the Faraday constant (96,485 C mol^−1^), the diffusion coefficient of H_2_ (3.7 × 10^−5^ cm^2^ s^−1^), the kinematic viscosity (1.01 × 10^−2^ cm^2^ s^−1^), the solubility of H_2_ (7.33 × 10^−4^ mol L^−1^), and the different rotation speeds, respectively.

Where n is the number of electrons involved in the HOR, F is the Faraday constant, D is the diffusion coefficient of the reactant, ν is the viscosity coefficient of electrolyte, C_0_ is the solubility of H_2_ in the electrolyte, ω is the rotating speed, and B is the Levich constant. The exchange current density (j_0_) was deduced from Butler–Volmer equation:

(4) $$j_{k}=j_{0}\left(e^{\frac{\alpha_{a}nF\eta }{RT}}-e^{-\frac{\alpha_{c}nF\eta }{RT}}\right)$$

where η is the overpotential, F is the Faraday constant, n is the number of electrons transferred (1), R is the universal gas constant, T is the Kelvin temperature, and α_a_ and α_c_ are transfer coefficients for the HOR and HER. The fitting was performed with the transfer coefficients α_a_ + α_c_ = 1.

In micro-polarization regions, the B-V equation can be expanded using Taylor’s formula and simplified to:

(5) $$j_{0}=\frac{j_{k}}{\eta }\frac{RT}{nF}$$

By linearly fitting the polarization curve in the micro-polarization region, the j_0_ can be obtained.

The electrochemically active surface area (ECSA) was estimated by measuring the double-layer capacitance (C_dl_) using cyclic voltammetry (CV) in the non-Faradaic potential region. All measurements were performed in 0.1 M KOH electrolyte within the respective non-Faradaic potential window, where no Faradaic current was observed. CV curves were recorded at various scan rates (v) of 2, 4, 6, 8, and 10 mV·s^−1^. The double-layer charging current ($\Delta j=j_{\mathrm{anodic}}-j_{\mathrm{cathodic}}$) was measured at the center of the potential window and plotted against the scan rate. The slope of the linear fit corresponds to C_dl_, according to the equation:

(6) $$\Delta j=C_{dl}\times v$$

The ECSA was then calculated using the equation:

(7) $$ECSA=\frac{C_{dl}}{C_{s}}$$

where C_s_ is the specific capacitance of the planar electrode surface. In 0.1 M KOH, the value of C_s_ was taken as 0.040 mF·cm^−2^.

### 3.9. DFT Calculations

DFT calculations were performed by the Vienna Ab initio Simulation Package (VASP) with the projector augmented wave method [29,30]. For the exchange and correlation energy density functions, the Perdew–Burke–Ernzerhof (PBE) generalized gradient approximation (GGA) was adopted [31,32]. For the cutoff energy, the 500 eV was chosen, which was consistent with previous work [33]. In order to improve the accuracy of model calculation, the DFT+U calculation method was used to describe the correlation of 4f rare earth metal elements. The U values of DFT+U are tested using the linear response approximation method (Figure S29, Supporting Information), and the calculated results show that the U values of Ce is 6.1 eV. Furthermore, due to the fully filled 4d10 shell electron configuration of Pd and its strong delocalization property, the Coulomb repulsion between electrons is relatively weak. Therefore, in most cases, standard GGA or meta-GGA functionals (such as PBE, PBEsol or SCAN) are sufficient to accurately describe its electronic states, and usually no additional U parameter needs to be introduced [34,35,36,37,38,39,40]. The 3 × 3 × 3 and 2 × 2 × 1 Monkhorst–Pack type k-point sampling was chosen to optimize bulk structure and slab model [41], choosing 0.01 eV/Å and 10^−4^ eV as the convergence criteria of force and energy, respectively.

The model consists of 84 Pd atoms, 60 O atoms and 30 Ce atoms. According to the TEM images, the main structure of the model is the interface heterostructure of Pd-CeO_2_. The exposed surfaces of both the Pd and CeO_2_ phases are of the 111 type.

## 4. Conclusions

In summary, we have successfully designed and synthesized a high-performance alkaline hydrogen oxidation reaction (HOR) electrocatalyst by constructing a well-defined Pd-CeO_2_ heterointerface on a nitrogen-doped carbon support. Comprehensive experimental characterizations combined with theoretical calculations reveal that the intimate interfacial contact induces spontaneous electron transfer from Pd to CeO_2_ via Pd-O-Ce bridges. This charge redistribution effectively modulates the electronic structure of Pd, notably downshifting its d-band center, which in turn optimizes the adsorption energies for the key reaction intermediates, *H and OH*.

The synergistic effect at the heterointerface leads to a dual optimization: it weakens the overly strong hydrogen binding on Pd while significantly enhancing the hydroxide adsorption at the interface. This breaks the conventional scaling relationship and substantially lowers the overall reaction energy barrier. As a result, the Pd-CeO_2_/NC catalyst exhibits exceptional HOR activity, with an exchange current density (3.66 mA cm^−2^) doubling that of commercial Pt/C, along with superior mass activity. More importantly, the catalyst demonstrates remarkable long-term stability and outstanding CO tolerance, maintaining high activity even in a 1000 ppm CO/H_2_ atmosphere, which addresses critical challenges for practical anion exchange membrane fuel cell (AEMFC) applications.

This work underscores the great potential of metal–oxide heterointerface engineering in regulating electronic structures for multi-intermediate electrocatalytic reactions. The fundamental insights gained—specifically on constructing active *OH adsorption sites and achieving an optimal balance between *H and *OH binding—provide a valuable design principle for developing next-generation, high-performance, and durable electrocatalysts for alkaline energy conversion technologies.

## Data Availability

The original contributions presented in this study are included in the article/Supplementary Materials. Further inquiries can be directed to the corresponding authors.

## Supplementary Materials

The following supporting information can be downloaded at: https://www.mdpi.com/article/10.3390/molecules31081306/s1, Figure S1. SEM images of NC. Figure S2. TEM image of CeO_2_/NC. Figure S3. XRD pattern of CeO_2_/NC. Figure S4. (a) TEM image of Pd/NC. (b) TEM image of Pd-CeO_2_/NC. (c) XRD pattern of Pd/NC. Figure S5. TEM images of CeO_2_/NC (5 mg). (b) TEM images of CeO_2_/NC (10 mg). (c) TEM images of CeO_2_/NC (15 mg). (d) TEM images of CeO_2_/NC (20 mg). Figure S6. (a) TEM images of Pd-CeO_2_(Big)/NC; (b) TEM images of Pd-CeO_2_/NC. Figure S7. HAADF and corresponding EDS elemental mapping of (a) Pd-CeO_2_(Big)/NC. (b) Pd-CeO_2_/NC. Figure S8. The line scanning EELS spectra of Pd-CeO_2_/NC cluster. Ce signals were not detected in the linear scan EELS spectra of the regions marked as 1, 3, and 4. Figure S9. HAADF and corresponding EDS elemental mapping of Pd-CeO_2_/NC. Figure S10. XPS survey spectra of (a) Pd/NC. (b) CeO_2_/NC. (c) Pd-CeO_2_/NC. Figure S11. C 1s XPS spectra of (a) Pd/NC. (b) CeO_2_/NC. (c) Pd-CeO_2_/NC. Figure S12. O 1s XPS spectra of (a) CeO_2_/NC. (b) Pd-CeO_2_/NC. Figure S13. k-space EXAFS fitting curve of Pd-CeO_2_/NC. Figure S14. (a) k-space fitting curve and (b) FT-EXAFS R-space fitting curve of Pd Foil. Figure S15. (a) XRD patterns of Pd-CeO_2_/NC(Ce:Pd = x). (b) Polarization curves of Pd-CeO_2_/NC(Ce:Pd = x) in H_2_-saturated 0.1 M KOH solution with the rotation rate of 1600 rpm and the scan rate of 5 mV s^−1^. The x in parentheses represents the molar ratio of Ce to Pd. Figure S16. Polarization curves of Pt/C, Pd-CeO_2_(Big)/NC, and Pd-CeO_2_/NC tested in H_2_-saturated 0.1 M KOH solution with the rotation rate of 1600 rpm and the scan rate of 5 mV s^−1^. Figure S17. Polarization curves of Pt/C, NC, CeO_2_/NC, Pd/NC, and Pd-CeO_2_/NC tested in H_2_-saturated 0.1 M KOH solution with the rotation rate of 1600 rpm and the scan rate of 5 mV s^−1^. The polarization curve has not undergone IR correction. Figure S18. Tafel plots of the catalysts where the current densities were normalized to their ECSA. Figure S19. Koutecky−Levich plot of Pd-CeO_2_/NC at an overpotential of 0.4 V. Figure S20. CV curves of catalysts with different sweep speeds. Figure S21. XRD patterns of Pd-CeO_2_/NC before and after stability test. Figure S22. Cyclic voltammetry curves of Pd-CeO_2_/NC before and after stability test. Figure S23. The atomic structure. (a) Pd (111). (b) CeO_2_. Figure S24. The DOS diagrams. (a) Pd (111). (b) Pd-CeO_2_/NC. Figure S25. Optimized atomic structure of (a) H adsorption, (b) H and OH adsorption, (d) H_2_O adsorption on Pd (111)/NC. The gray, pink and white spheres represent Ce, O and H atoms, respectively. Figure S26. Optimized atomic structure of (a) H adsorption, (b) H and OH adsorption, (d) H_2_O adsorption on CeO_2_/NC. The cyan, pink and white spheres represent Ce, O and H atoms, respectively. Figure S27. Optimized atomic structure of (a) H adsorption, (b) H and OH adsorption, (d) H_2_O adsorption on Pd-CeO_2_/NC. The gray, cyan, pink and white spheres represent Pd, Ce, O and H atoms, respectively. Figure S28. H adsorption energy of Pd-CeO_2_/NC and Pd (111)/NC. Figure S29. Linear response curve for the U-value test of Ce. Table S1. EXAFS fitting parameters at the Pd K-edge for various samples [42]. Table S2. Chemical compositions and ICP-OES results for different catalysts. Table S3. Comparison of the intrinsic HOR activity of our catalysts and other state-of-the-art HOR catalysts [43,44,45,46,47,48,49,50,51,52,53,54,55].

## References

[1] Zou X., Zhang Y. (2015). Noble Metal-Free Hydrogen Evolution Catalysts for Water Splitting. Chem. Soc. Rev..

[2] Zhang S., Ren R., Cao J., Zhang D., Bai J., Han C., Xiao L., Zhuang L., Song P., Xu W. (2025). Ru-MnO Heterostructure Clusters Toward Efficient and CO-Tolerant Alkaline Hydrogen Oxidation Reaction. Adv. Energy Mater..

[3] Odenweller A., Ueckerdt F., Nemet G.F., Jensterle M., Luderer G. (2022). Probabilistic Feasibility Space of Scaling up Green Hydrogen Supply. Nat. Energy.

[4] Ramaswamy N., Ghoshal S., Bates M.K., Jia Q., Li J., Mukerjee S. (2017). Hydrogen Oxidation Reaction in Alkaline Media: Relationship between Electrocatalysis and Electrochemical Double-Layer Structure. Nano Energy.

[5] Setzler B.P., Zhuang Z., Wittkopf J.A., Yan Y. (2016). Activity Targets for Nanostructured Platinum-Group-Metal-Free Catalysts in Hydroxide Exchange Membrane Fuel Cells. Nat. Nanotechnol..

[6] Zhang X., Xia L., Zhao G., Zhang B., Chen Y., Chen J., Gao M., Jiang Y., Liu Y., Pan H. (2023). Fast and Durable Alkaline Hydrogen Oxidation Reaction at the Electron-Deficient Ruthenium–Ruthenium Oxide Interface. Adv. Mater..

[7] Fang J., Wang H., Dang Q., Wang H., Wang X., Pei J., Xu Z., Chen C., Zhu W., Li H. (2024). Atomically Dispersed Iridium on Mo_2_C as an Efficient and Stable Alkaline Hydrogen Oxidation Reaction Catalyst. Nat. Commun..

[8] Strmcnik D., Uchimura M., Wang C., Subbaraman R., Danilovic N., van der Vliet D., Paulikas A.P., Stamenkovic V.R., Markovic N.M. (2013). Improving the Hydrogen Oxidation Reaction Rate by Promotion of Hydroxyl Adsorption. Nat. Chem..

[9] McCrum I.T., Koper M.T.M. (2020). The Role of Adsorbed Hydroxide in Hydrogen Evolution Reaction Kinetics on Modified Platinum. Nat. Energy.

[10] Rheinländer P.J., Herranz J., Durst J., Gasteiger H.A. (2014). Kinetics of the Hydrogen Oxidation/Evolution Reaction on Polycrystalline Platinum in Alkaline Electrolyte Reaction Order with Respect to Hydrogen Pressure. J. Electrochem. Soc..

[11] Su L., Zhao Y., Jin Y., Liu Z., Cui H., Luo W. (2022). Identifying the Role of Hydroxyl Binding Energy in a Non-Monotonous Behavior of Pd-Pd_4_S for Hydrogen Oxidation Reaction. Adv. Funct. Mater..

[12] Valdés-López V.F., Mason T., Shearing P.R., Brett D.J.L. (2020). Carbon Monoxide Poisoning and Mitigation Strategies for Polymer Electrolyte Membrane Fuel Cells—A Review. Prog. Energy Combust. Sci..

[13] Su L., Wu H., Zhang S., Cui C., Zhou S., Pang H. (2025). Insight Into Intermediate Behaviors and Design Strategies of Platinum Group Metal-Based Alkaline Hydrogen Oxidation Catalysts. Adv. Mater..

[14] Li H.-C., Zhang Y.-J., Hu X., Liu W.-J., Chen J.-J., Yu H.-Q. (2018). Metal–Organic Framework Templated Pd@PdO–Co_3_O_4_ Nanocubes as an Efficient Bifunctional Oxygen Electrocatalyst. Adv. Energy Mater..

[15] Zhang X., Hui L., He F., Li Y. (2025). The Interfacial Interpenetration Effect for Controlled Reaction Stability of Palladium Catalysts. J. Am. Chem. Soc..

[16] Johnson A.D., Daley S.P., Utz A.L., Ceyer S.T. (1992). The Chemistry of Bulk Hydrogen: Reaction of Hydrogen Embedded in Nickel with Adsorbed CH_3_. Science.

[17] Feng Y., Guan Y., Zhou E., Zhang X., Wang Y. (2022). Nanoscale Double-Heterojunctional Electrocatalyst for Hydrogen Evolution. Adv. Sci..

[18] Bae C., Ho T.A., Kim H., Lee S., Lim S., Kim M., Yoo H., Montero-Moreno J.M., Park J.H., Shin H. (2017). Bulk Layered Heterojunction as an Efficient Electrocatalyst for Hydrogen Evolution. Sci. Adv..

[19] Li Z., Hu M., Wang P., Liu J., Yao J., Li C. (2021). Heterojunction Catalyst in Electrocatalytic Water Splitting. Coord. Chem. Rev..

[20] Zhao T., Li M., Xiao D., Yang X., An L., Deng Z., Shen T., Gong M., Chen Y., Liu H. (2024). Improving Alkaline Hydrogen Oxidation through Dynamic Lattice Hydrogen Migration in Pd@Pt Core-Shell Electrocatalysts. Angew. Chem. Int. Ed..

[21] Guo Z., Cui Y., Liu W. (2024). High-Performance and Durable Pd_5_P_2_/PdP_2_ Heterointerface for All-pH Hydrogen Evolution Reactions. ACS Catal..

[22] Ke X., Zhou F., Chen Y., Zhao M., Yang Y., Jin H., Dong Y., Zou C., Chen X., Zhang L. (2023). Modifying Charge Transfer between Rhodium and Ceria for Boosted Hydrogen Oxidation Reaction in Alkaline Electrolyte. J. Colloid Interface Sci..

[23] Wang H., Wang X., Gao F., Chen J., Ren X., Shen Z., Wang K., Qi F., Liu Y., Gao Y. (2026). Synergistic Catalysis of Pt-Based High-Entropy Clusters Coupled with Super-Hydrophilic CeO_2_ Enables Efficient Anion Exchange Membrane Water Electrolysis. Adv. Mater..

[24] Wu Z., Yang P., Li Q., Xiao W., Li Z., Xu G., Liu F., Jia B., Ma T., Feng S. (2023). Microwave Synthesis of Pt Clusters on Black TiO_2_ with Abundant Oxygen Vacancies for Efficient Acidic Electrocatalytic Hydrogen Evolution. Angew. Chem. Int. Ed..

[25] Sheng W., Gasteiger H.A., Shao-Horn Y. (2010). Hydrogen Oxidation and Evolution Reaction Kinetics on Platinum: Acid vs. Alkaline Electrolytes. J. Electrochem. Soc..

[26] Li J., Qiu R., Zhang S., Peng L., Dong Y., Jiang Y., Li Y., Fang N., Yu J., Dong J.-C. (2025). Synergistically Enhanced Co-Adsorption of Reactant and Hydroxyl on Platinum-Modified Copper Oxide for High-Performance HMF Oxidation. Adv. Mater..

[27] Su L., Chen J., Yang F., Li P., Jin Y., Luo W., Chen S. (2023). Electric-Double-Layer Origin of the Kinetic pH Effect of Hydrogen Electrocatalysis Revealed by a Universal Hydroxide Adsorption-Dependent Inflection-Point Behavior. J. Am. Chem. Soc..

[28] Men Y., Su X., Li P., Tan Y., Ge C., Jia S., Li L., Wang J., Cheng G., Zhuang L. (2022). Oxygen-Inserted Top-Surface Layers of Ni for Boosting Alkaline Hydrogen Oxidation Electrocatalysis. J. Am. Chem. Soc..

[29] Kresse G., Furthmüller J. (1996). Efficiency of Ab-Initio Total Energy Calculations for Metals and Semiconductors Using a Plane-Wave Basis Set. Comput. Mater. Sci..

[30] Kresse G., Furthmüller J. (1996). Efficient Iterative Schemes for Ab Initio Total-Energy Calculations Using a Plane-Wave Basis Set. Phys. Rev. B.

[31] Kresse G., Joubert D. (1999). From Ultrasoft Pseudopotentials to the Projector Augmented-Wave Method. Phys. Rev. B.

[32] Blöchl P.E. (1994). Projector Augmented-Wave Method. Phys. Rev. B.

[33] Rossmeisl J., Logadottir A., Nørskov J.K. (2005). Electrolysis of Water on (Oxidized) Metal Surfaces. Chem. Phys..

[34] Wang L., Deo S., Mukhopadhyay A., Pantelis N.A.I., Janik M.J., Rioux R.M. (2022). Emergent Behavior in Oxidation Catalysis over Single-Atom Pd on a Reducible CeO_2_ Support via Mixed Redox Cycles. ACS Catal..

[35] Kim Y., Lee H., Kwak J.H. (2020). Mechanism of CO Oxidation on Pd/CeO_2_(100): The Unique Surface-Structure of CeO_2_(100) and the Role of Peroxide. ChemCatChem.

[36] Mehar V., Kim M., Shipilin M., Van den Bossche M., Gustafson J., Merte L.R., Hejral U., Grönbeck H., Lundgren E., Asthagiri A. (2018). Understanding the Intrinsic Surface Reactivity of Single-Layer and Multilayer PdO(101) on Pd(100). ACS Catal..

[37] Pothu R., Mitta H., Banerjee P., Boddula R., Srivastava R.K., Kalambate P.K., Naik R., Bahgat Radwan A., Al-Qahtani N. (2023). Insights into the Influence of Pd Loading on CeO_2_ Catalysts for CO_2_ Hydrogenation to Methanol. Mater. Sci. Energy Technol..

[38] Colussi S., Fornasiero P., Trovarelli A. (2020). Structure-Activity Relationship in Pd/CeO_2_ Methane Oxidation Catalysts. Chin. J. Catal..

[39] Wang M., Ma P., Wu Z., Chu S., Zheng Y., Zhou Z., Weng W. (2022). Evolution of Pd Chemical States and Effects of C_3_H_6_ and H_2_O on the CO Oxidation over Pd/CeO_2_ Catalyst. Appl. Surf. Sci..

[40] Chadi D.J. (1977). Spin-Orbit Splitting in Crystalline and Compositionally Disordered Semiconductors. Phys. Rev. B.

[41] Henkelman G., Arnaldsson A., Jónsson H. (2006). A Fast and Robust Algorithm for Bader Decomposition of Charge Density. Comput. Mater. Sci..

[42] Zhang W., Hao X., Liu X., Chu M., Li S., Wang X., Jiang F., Wang L., Zhang Q., Chen J. (2025). Photocatalytic Conversion of Polyester-Derived Alcohol into Value-Added Chemicals by Engineering Atomically Dispersed Pd Catalyst. Angew. Chem. Int. Ed..

[43] Zhou Y., Xie Z., Jiang J., Wang J., Song X., He Q., Ding W., Wei Z. (2020). Lattice-Confined Ru Clusters with High CO Tolerance and Activity for the Hydrogen Oxidation Reaction. Nat. Catal..

[44] Zhao T., Wang G., Gong M., Xiao D., Chen Y., Shen T., Lu Y., Zhang J., Xin H., Li Q. (2020). Self-Optimized Ligand Effect in L12-PtPdFe Intermetallic for Efficient and Stable Alkaline Hydrogen Oxidation Reaction. ACS Catal..

[45] Shi H., Yang Y., Meng P., Yang J., Zheng W., Wang P., Zhang Y., Chen X., Cheng Z., Zong C. (2024). Local Charge Transfer Unveils Antideactivation of Ru at High Potentials for the Alkaline Hydrogen Oxidation Reaction. J. Am. Chem. Soc..

[46] Han L., Ou P., Liu W., Wang X., Wang H.-T., Zhang R., Pao C.-W., Liu X., Pong W.-F., Song J. (2022). Design of Ru-Ni Diatomic Sites for Efficient Alkaline Hydrogen Oxidation. Sci. Adv..

[47] Xiao W., Lei W., Wang J., Gao G., Zhao T., Cordeiro M.A.L., Lin R., Gong M., Guo X., Stavitski E. (2018). Tuning the Electrocatalytic Activity of Pt by Structurally Ordered PdFe/C for the Hydrogen Oxidation Reaction in Alkaline Media. J. Mater. Chem. A.

[48] Zhao T., Hu Y., Gong M., Lin R., Deng S., Lu Y., Liu X., Chen Y., Shen T., Hu Y. (2020). Electronic Structure and Oxophilicity Optimization of Mono-Layer Pt for Efficient Electrocatalysis. Nano Energy.

[49] Shen D., Sun F., Liang Z., Mei B., Xie Y., Wang Y., Wang L., Fu H. (2025). Oxygen Spillover on Supported Pt-Cluster for Anti-CO-Poisoning Hydrogen Oxidation. Nat. Commun..

[50] Zhao Y., Wu D., Luo W. (2022). Correlating Alkaline Hydrogen Electrocatalysis and Hydroxide Binding Energies on Mo-Modified Ru Catalysts. ACS Sustain. Chem. Eng..

[51] Xue Y., Shi L., Liu X., Fang J., Wang X., Setzler B.P., Zhu W., Yan Y., Zhuang Z. (2020). A Highly-Active, Stable and Low-Cost Platinum-Free Anode Catalyst Based on RuNi for Hydroxide Exchange Membrane Fuel Cells. Nat. Commun..

[52] Cui Z., Ren Z., Ma C., Chen B., Chen G., Lu R., Zhu W., Gan T., Wang Z., Zhuang Z. (2024). Dilute RuCo Alloy Synergizing Single Ru and Co Atoms as Efficient and CO-Resistant Anode Catalyst for Anion Exchange Membrane Fuel Cells. Angew. Chem. Int. Ed..

[53] Wu J., Gao X., Liu G., Qiu X., Xia Q., Wang X., Zhu W., He T., Zhou Y., Feng K. (2024). Immobilizing Ordered Oxophilic Indium Sites on Platinum Enabling Efficient Hydrogen Oxidation in Alkaline Electrolyte. J. Am. Chem. Soc..

[54] Wang P., Yang Y., Zheng W., Cheng Z., Wang C., Chen S., Wang D., Yang J., Shi H., Meng P. (2023). V–O Species-Doped Carbon Frameworks Loaded with Ru Nanoparticles as Highly Efficient and CO-Tolerant Catalysts for Alkaline Hydrogen Oxidation. J. Am. Chem. Soc..

[55] Cai B., Shen D., Xie Y., Yan H., Wang Y., Chen X., Wang L., Fu H. (2024). Unlocking Superior Hydrogen Oxidation and CO Poisoning Resistance on Pt Enabled by Tungsten Nitride-Mediated Electronic Modulation. J. Am. Chem. Soc..

