# Supplementary material for: Electronic Modulation via a Pd-CeO2 Heterointerface for Superior Alkaline Hydrogen Oxidation
Source: Molecules. 2026 Apr 17;31(8):1306. doi: 10.3390/molecules31081306 (PMC13118421; doi:10.3390/molecules31081306)
Supplement: Supplementary file 1 [file molecules-31-01306-s001.zip › molecules-4221130-supplementary.pdf]

Supporting Information

Electronic Modulation via a Pd–CeO_2 Heterointerface for Superior Alkaline Hydrogen Oxidation

Minhui Zhong [1], Qingzhen Xu [1], Wenhai Xu [1], Wei Zhang [1], Man Zhao [1,*], Yizhe Li [2,*] and Wen Liu [1,*]

[1] Department of Chemistry, Beijing University of Chemical Technology, Beijing 100029, China; 2024210893@buct.edu.cn (M. Z.) ; xuqingzhen1208@163.com (Q. X.) ; 2025400370@buct.edu.cn (W. X.) ; 2024400344@buct.edu.cn (W. Z)

[2] CAS Key Laboratory of Nanosystem and Hierarchical Fabrication, National Center for Nanoscience and Technology, Beijing 100190, China

* Correspondence: wenliu@mail.buct.edu.cn (W. L) ; liyz2025@nanoctr.cn (Y. L) ; zhaom@buct.edu.cn (M. Z.)

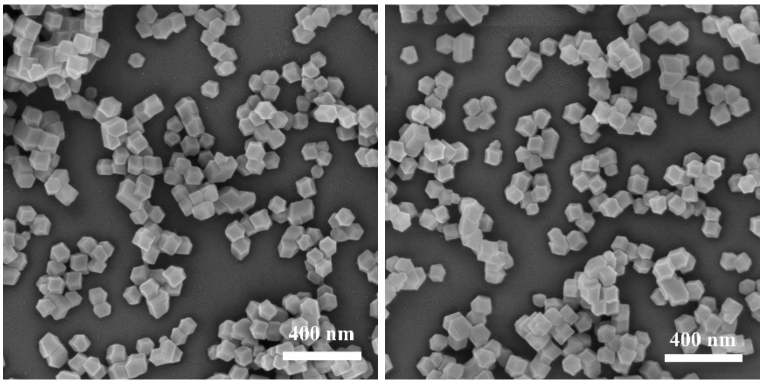

Figure S1. SEM images of NC

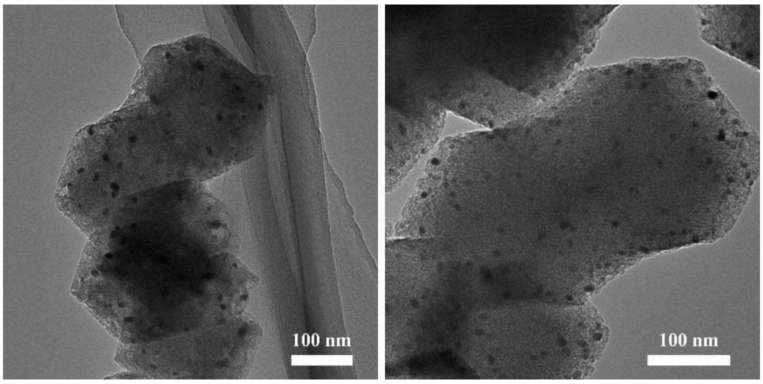

Figure S2. TEM image of CeO_2/NC.

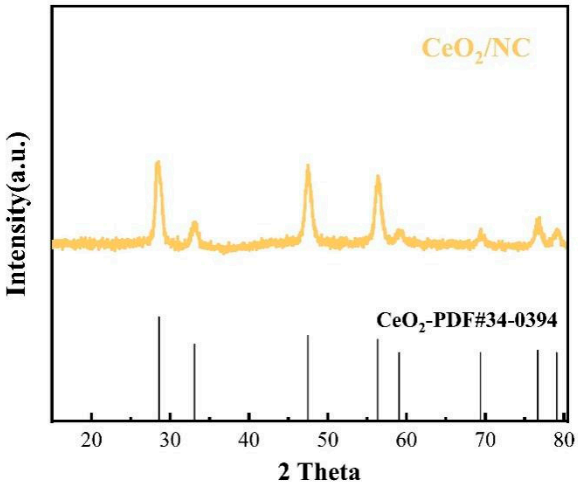

Figure S3. XRD pattern of CeO_2/NC.

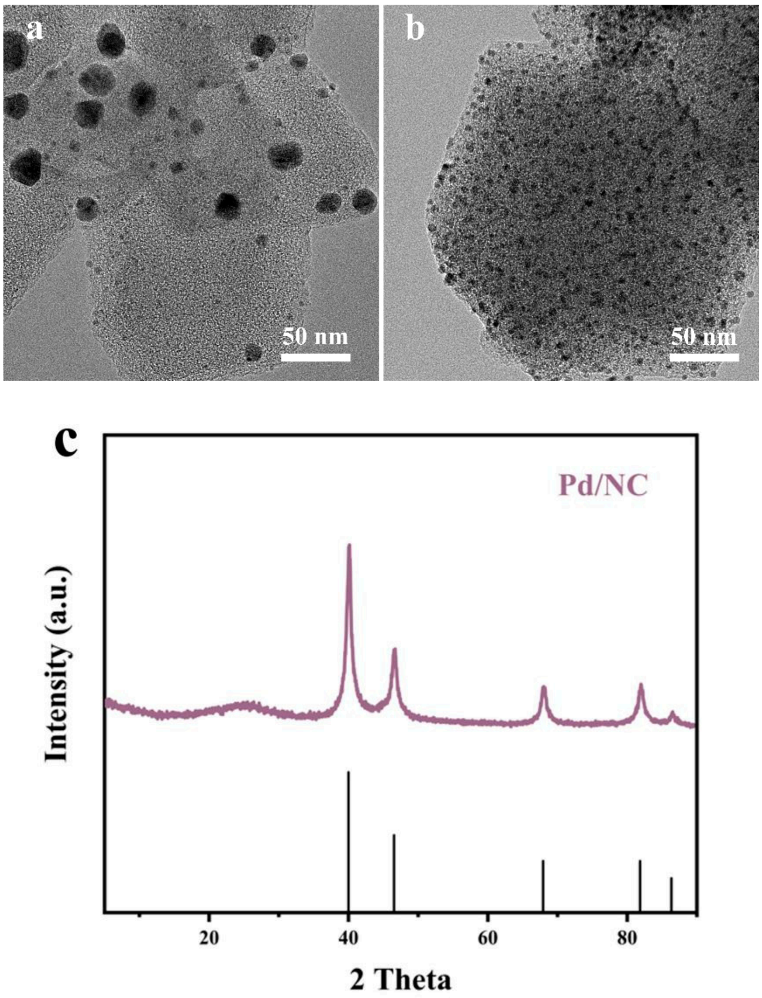

Figure S4. (a) TEM image of Pd/NC. (b) TEM image of Pd-CeO_2/NC. (c) XRD pattern of Pd/NC.

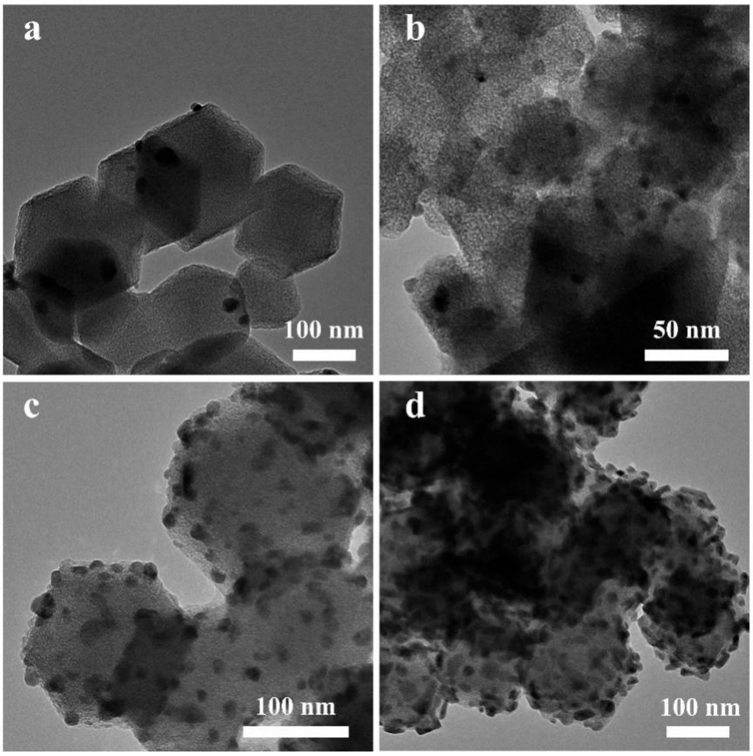

Figure S5. TEM images of CeO_2/NC(5 mg). (b) TEM images of CeO_2/NC (10 mg). (c) TEM images of CeO_2/NC (15 mg). (d) TEM images of CeO_2/NC (20 mg). The quality marked in parentheses represents the addition amount of cerium acetylacetone.

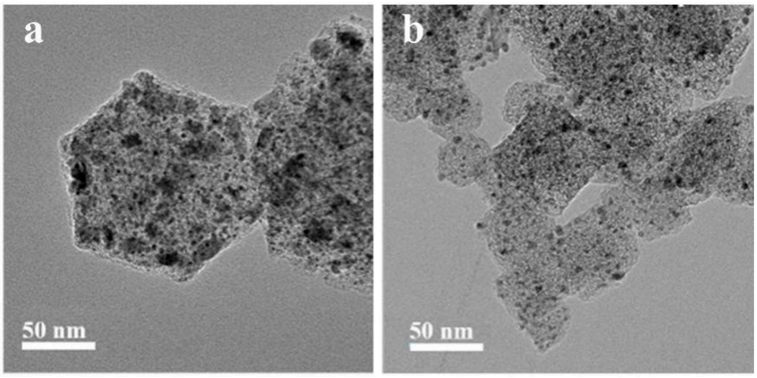

Figure S6. (a) TEM images of Pd-CeO_2(Big)/NC; (b) TEM images of Pd-CeO_2/NC.

During the synthesis process, Pd-CeO_2/NC utilizes $PdNO_3$ dissolved in strong acid as the source of Pd, whereas Pd-CeO_2(Big)/NC employs $PdCl_2$ in neutral conditions as the source of Pd.

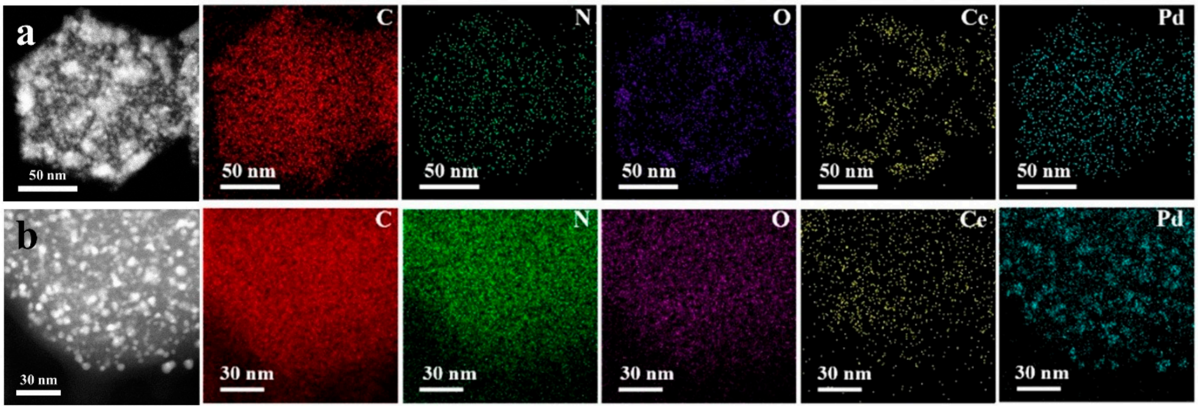

Figure S7. HAADF and corresponding EDS elemental mapping of (a) Pd-CeO_2(Big)/NC. (b) Pd-CeO_2/NC.

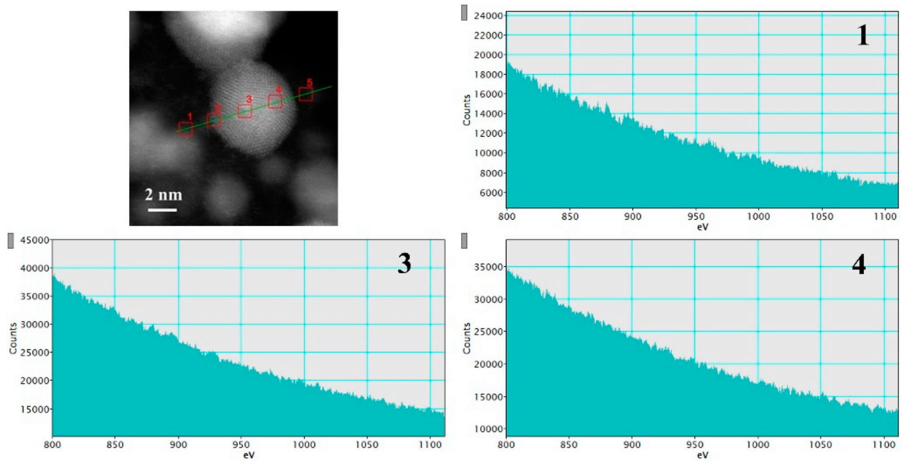

Figure S8. The line scanning EELS spectra of Pd-CeO_2/NC cluster. Ce signals were not detected in the linear scan EELS spectra of the regions marked as 1, 3, and 4.

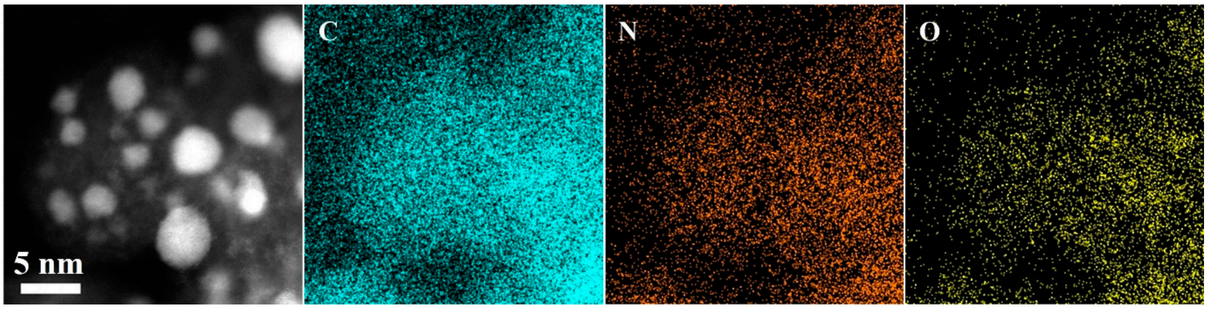

Figure S9. HAADF and corresponding EDS elemental mapping of Pd-CeO_2/NC.

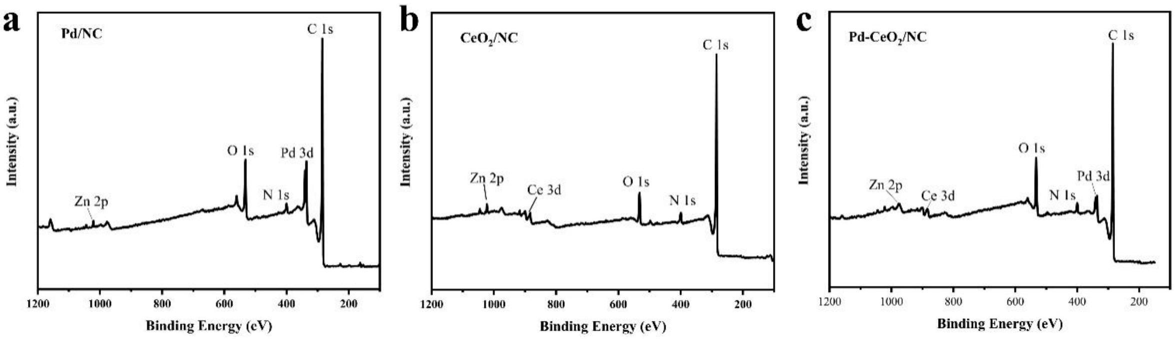

Figure S10. XPS survey spectra of (a) Pd/NC. (b) CeO_2/NC. (c) Pd-CeO_2/NC.

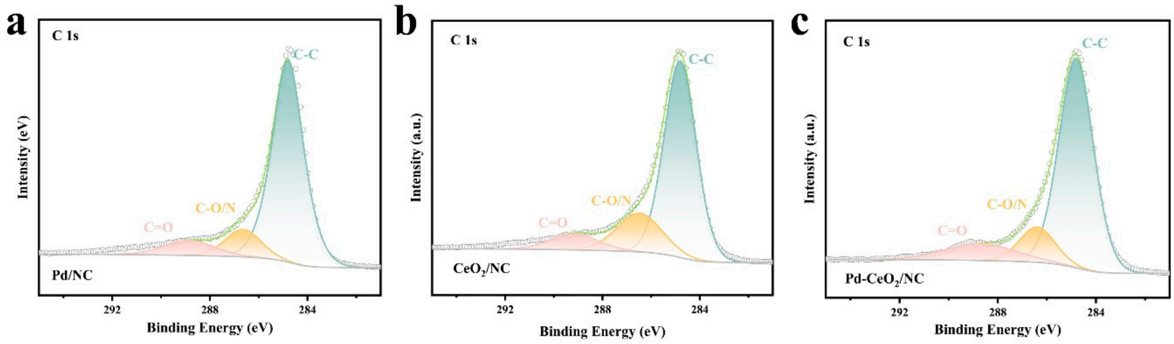

Figure S11. C 1s XPS spectra of (a) Pd/NC. (b) CeO_2/NC. (c) Pd-CeO_2/NC.

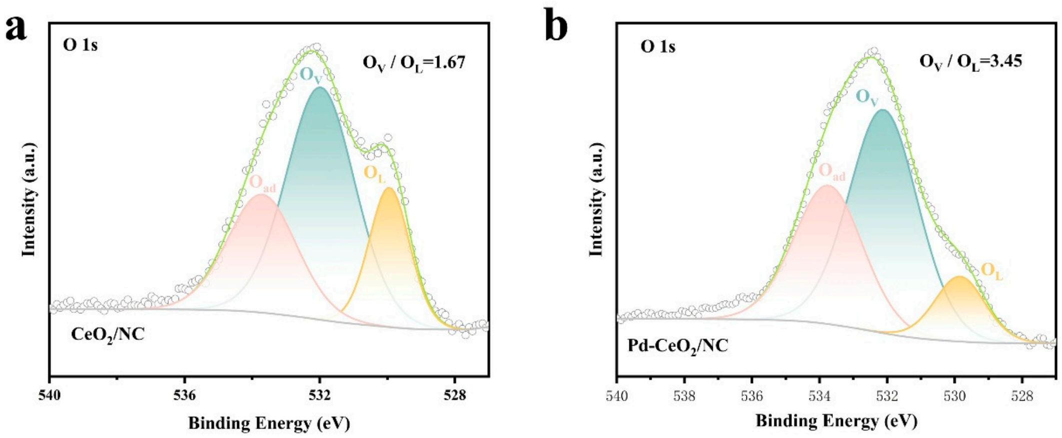

Figure S12. O 1s XPS spectra of (a) CeO_2/NC. (b) Pd-CeO_2/NC.

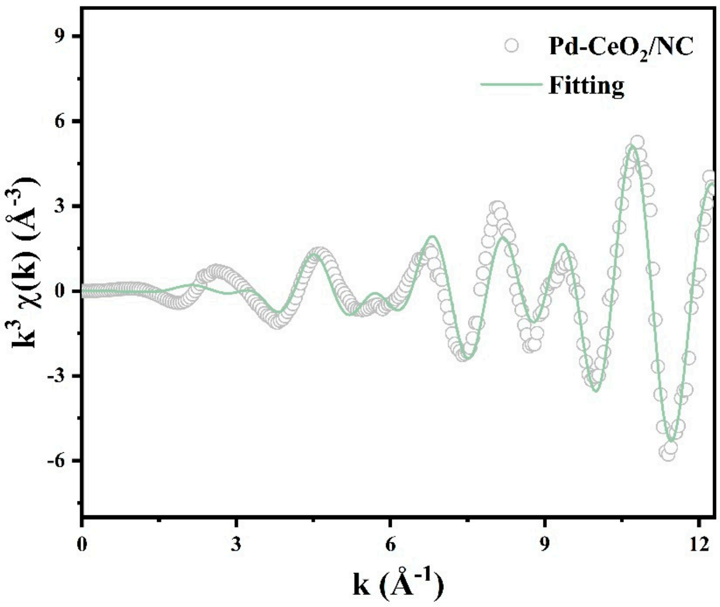

Figure S13. k-space EXAFS fitting curve of Pd-CeO_2/NC.

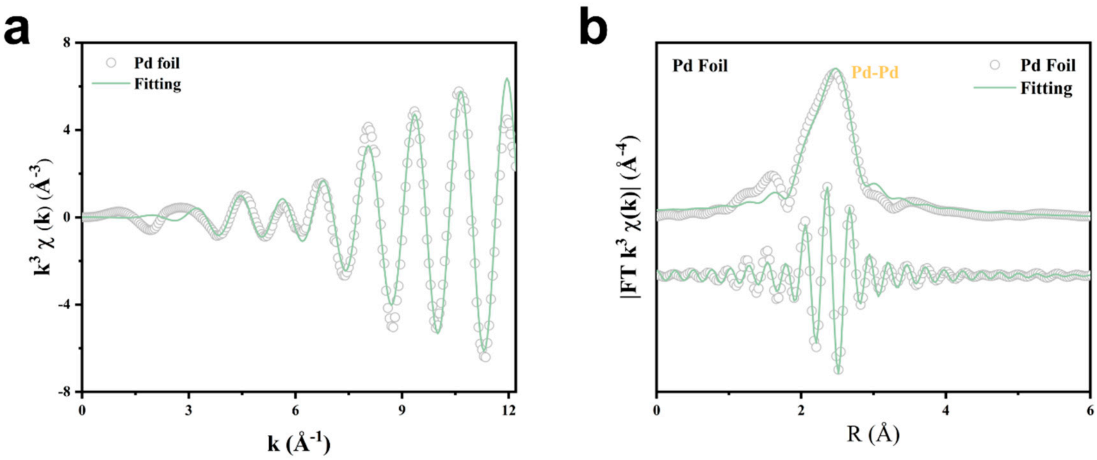

Figure S14. (a) k-space fitting curve and (b) FT-EXAFS R-space fitting curve of Pd Foil.

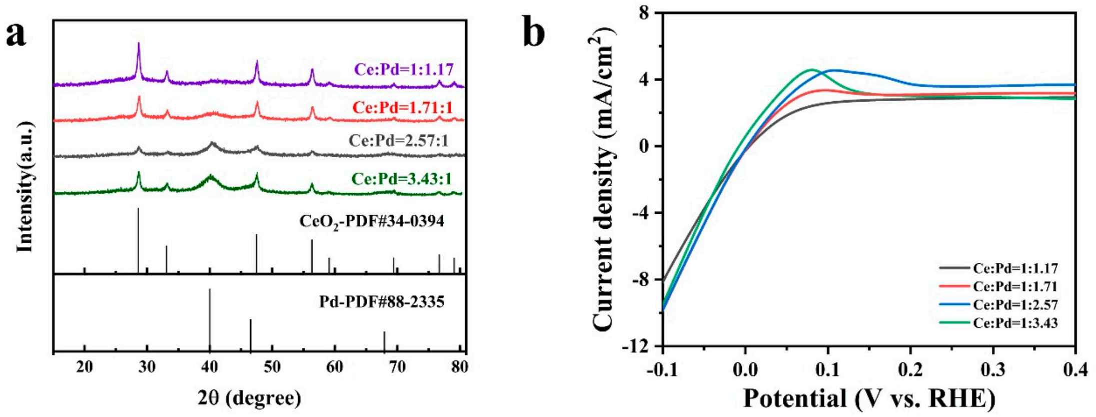

Figure S15. (a) XRD patterns of Pd-CeO_2/NC(Ce:Pd = x). (b) Polarization curves of Pd-CeO_2/NC(Ce:Pd = x) in H_2-saturated 0.1 M KOH solution with the rotation rate of 1600 rpm and the scan rate of 5 mV s^{-1}. The x in parentheses represents the molar ratio of Ce to Pd.

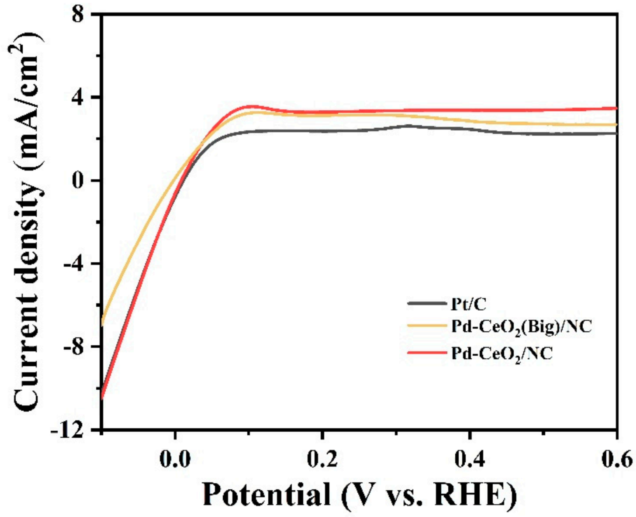

Figure S16. Polarization curves of Pt/C, Pd-CeO_2(Big)/NC, and Pd-CeO_2/NC tested in H_2-saturated 0.1 M KOH solution with the rotation rate of 1600 rpm and the scan rate of 5 mV s^{-1}.

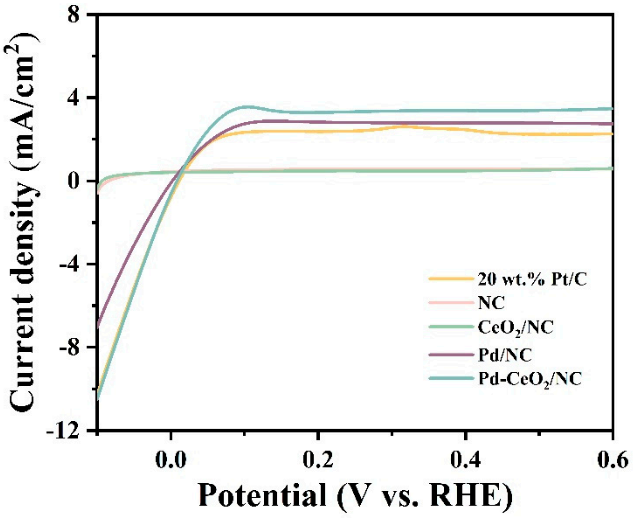

Figure S17. Polarization curves of Pt/C, NC, CeO_2/NC, Pd/NC, and Pd-CeO_2/NC tested in H_2-saturated 0.1 M KOH solution with the rotation rate of 1600 rpm and the scan rate of 5 mV s^{-1}. The polarization curve has not undergone IR correction.

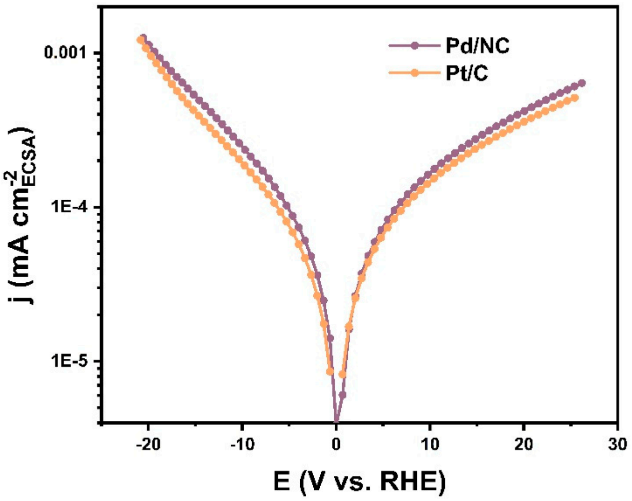

Figure S18. Tafel plots of the catalysts where the current densities were normalized to their ECSA.

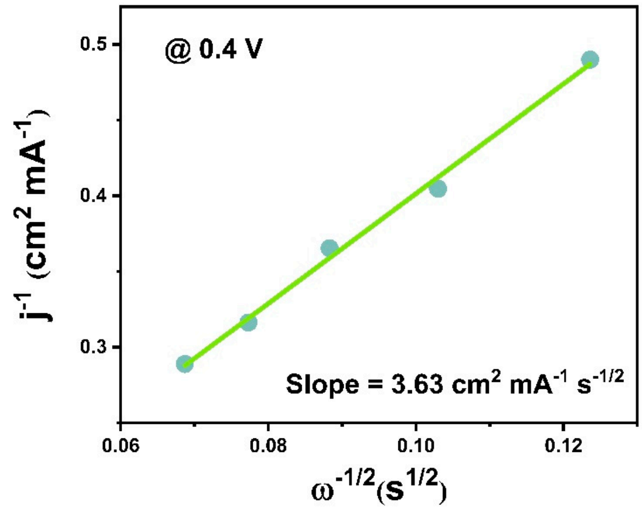

Figure S19. Koutecky−Levich plot of Pd-CeO_2/NC at an overpotential of 0.4 V.

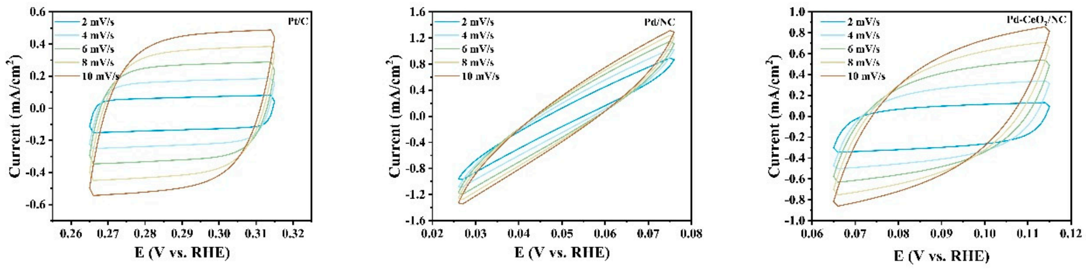

Figure S20. CV curves of catalysts with different sweep speeds.

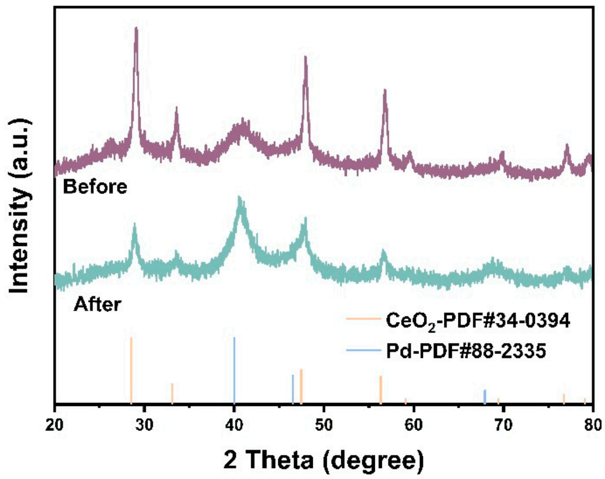

Figure S21. XRD patterns of Pd-CeO_2/NC before and after stability test.

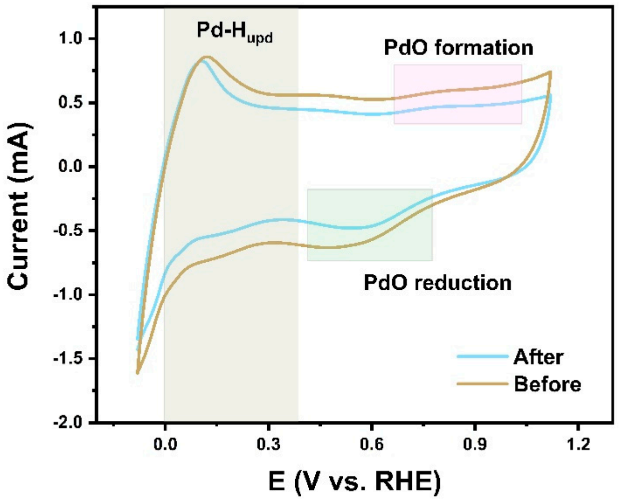

Figure S22. Cyclic voltammetry curves of Pd-CeO_2/NC before and after stability test.

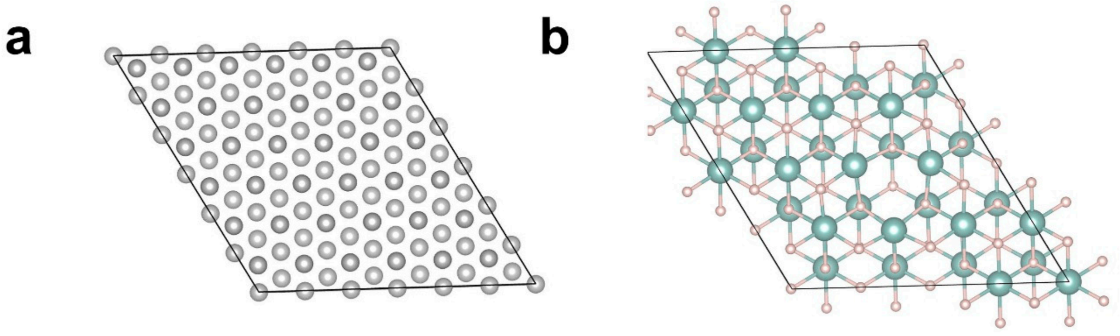

Figure S23. The atomic structure. (a) Pd (111). (b) CeO_2.

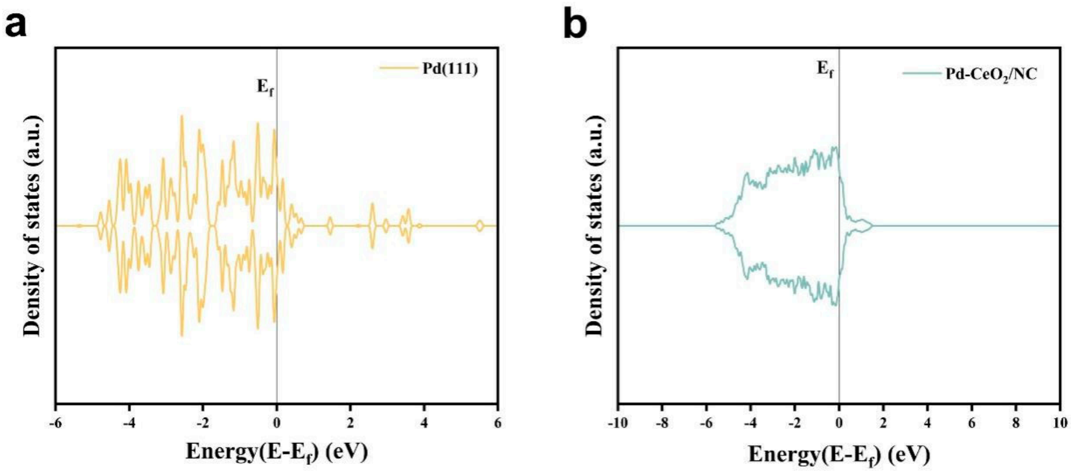

Figure S24. The DOS diagrams. (a) Pd(111). (b) Pd-CeO_2/NC.

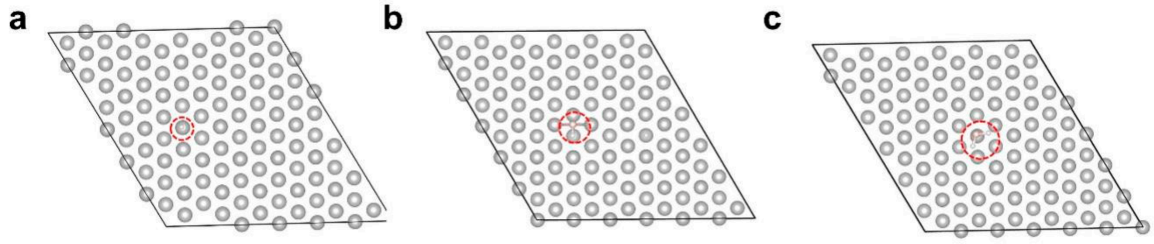

Figure S25. Optimized atomic structure of (a) H adsorption, (b) H and OH adsorption, (d) H_2O adsorption on Pd(111)/NC. The gray, pink and white spheres represent Ce, O and H atoms, respectively.

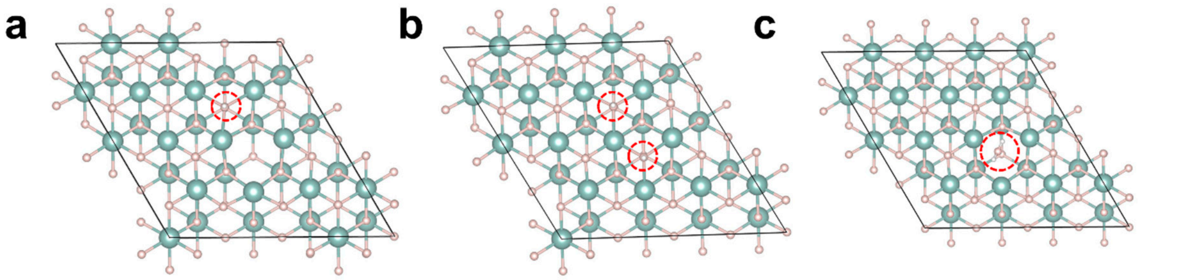

Figure S26. Optimized atomic structure of (a) H adsorption, (b) H and OH adsorption, (d) H_2O adsorption on CeO_2/NC. The cyan, pink and white spheres represent Ce, O and H atoms, respectively.

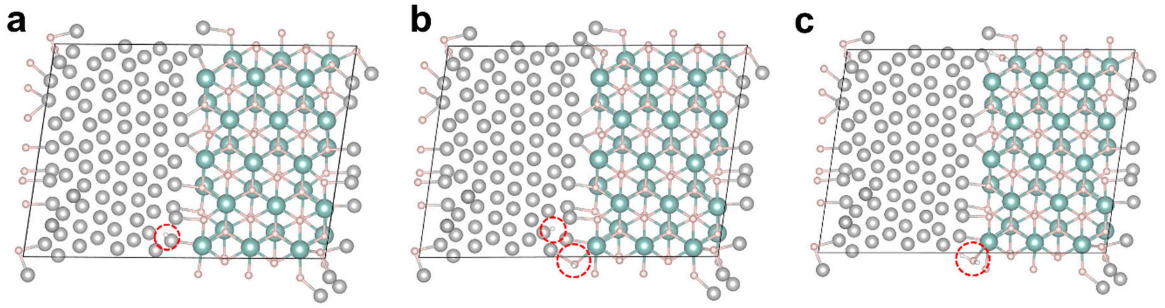

Figure S27. Optimized atomic structure of (a) H adsorption, (b) H and OH adsorption, (d) H_2O adsorption on Pd-CeO_2/NC. The gray, cyan, pink and white spheres represent Pd, Ce, O and H atoms, respectively.

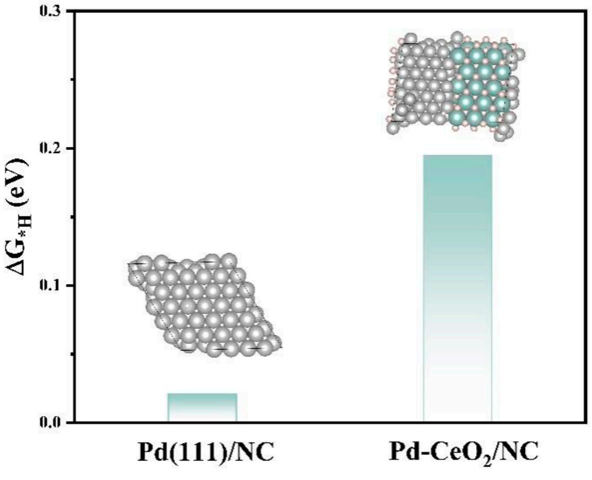

Figure S28. H adsorption energy of Pd-CeO_2/NC and Pd(111)/NC.

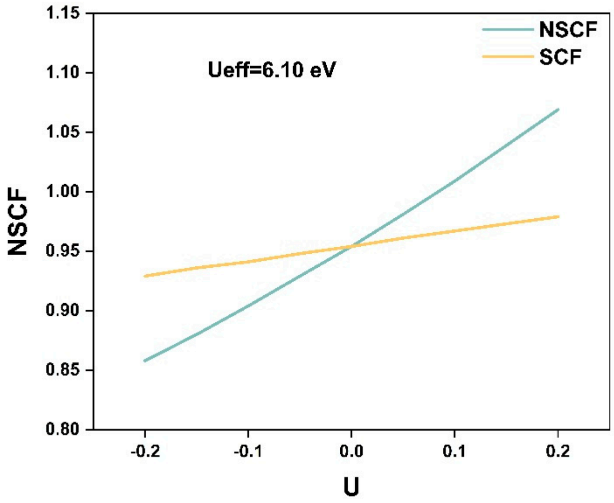

Figure S29. Linear response curve for the U-value test of Ce.

Table S1. EXAFS fitting parameters at the Pd K-edge for various samples.

Sample	Path	C.N.	R (Å)	$\sigma^2\times10^3$ (Å^2)	ΔE_0 (eV)	R-factor
Pd Foil	Pd-Pd	12*	2.74±0.01	5.3±0.2	5.4±0.3	0.002
$Pd\text{-}CeO_2/NC$	Pd-O	3.8±0.4	2.02±0.01	1.3±1.3	6.9±1.1	0.019
	Pd-Pd	5.8±1.3	3.02±0.01	4.2±3.5		
PdO[[1]]	Pd-O	4	2.02±0.01	1.2±1.3	-2.1±1.6	0.021
	Pd-Pd	4	3.05±0.01	4.1±1.2		
	Pd-Pd	8	3.45±0.01	7.2±1.4		

S_0^2 was set to 0.816; CN is the coordination number; R is the interatomic distance between absorber and backscatter atoms; σ^2 is Debye-Waller factor to account for both thermal and structural disorders; ΔE_0 is the inner potential correction; R factor is used to value the goodness of fit. An E_0 value of 24350.0 eV was used to calibrate all data with respect to the first inflection point of the absorption K-edge of Pd foil.

Table S2. Chemical compositions and ICP-OES results different catalyst.

Sample	Pd (wt. %)	Ce (wt. %)	Pd/Ce molar ration
Pd/NC	16.81	0	-
$Pd\text{-}CeO_2/NC$	13.60	4.74	2.87
CeO_2/NC	0	9.83	0

Table S3. Comparison of the intrinsic HOR activity of our catalysts and other state-of-the-art HOR catalysts.

**Catalyst**	**j_0 (mA cm^{-2})**	**Ref.**
**Pd-CeO_2/NC**	**3.66**	**This work**
**Pd/NC**	**1.83**	
**Pt/C**	**1.87**	
Ru@TiO_2	2.00	[[2]]
$(Pt_{0.9}Pd_{0.1})_3Fe/C$	1.56	[[3]]
$Pt_{0.9}Pd_{0.1}/C$	1.32	
$Mn_1O_x(OH)_y$@Ru/C	2.05	[[4]]
RuNi/NC	2.69	[[5]]
O-PdFe@Pt/C	1.24	[[6]]
Pd_3Co@Pt/C	1.28	[[7]]
H-Pt-W_3O/WC	3.68	[[8]]
Mo-Ru-2/C	3.25	[[9]]
Ru_7Ni_3/C	1.04	[[10]]
$(RuCo)_{NC+SAs}$/N-CNT	2.62	[[11]]
O-Pt_3In/ rGO	3.03	[[12]]
Ru/VOC	3.06	[[13]]
Pt@WN/rGO	3.94	[[14]]

Reference

[1] Zhang, W.; Hao, X.; Liu, X.; Chu, M.; Li, S.; Wang, X.; Jiang, F.; Wang, L.; Zhang, Q.; Chen, J.; Wang, D.; Cao, M. Photocatalytic Conversion of Polyester-Derived Alcohol into Value-Added Chemicals by Engineering Atomically Dispersed Pd Catalyst. *Angewandte Chemie International Edition* **2025**, *64* (18), e202500814. https://doi.org/10.1002/anie.202500814.

[2] Zhou, Y.; Xie, Z.; Jiang, J.; Wang, J.; Song, X.; He, Q.; Ding, W.; Wei, Z. Lattice-Confined Ru Clusters with High CO Tolerance and Activity for the Hydrogen Oxidation Reaction. *Nat Catal* **2020**, *3* (5), 454–462. https://doi.org/10.1038/s41929-020-0446-9.

[3] Zhao, T.; Wang, G.; Gong, M.; Xiao, D.; Chen, Y.; Shen, T.; Lu, Y.; Zhang, J.; Xin, H.; Li, Q.; Wang, D. Self-Optimized Ligand Effect in L12-PtPdFe Intermetallic for Efficient and Stable Alkaline Hydrogen Oxidation Reaction. *ACS Catal.* **2020**, *10* (24), 15207–15216. https://doi.org/10.1021/acscatal.0c03938.

[4] Shi, H.; Yang, Y.; Meng, P.; Yang, J.; Zheng, W.; Wang, P.; Zhang, Y.; Chen, X.; Cheng, Z.; Zong, C.; Wang, D.; Chen, Q. Local Charge Transfer Unveils Antideactivation of Ru at High Potentials for the Alkaline Hydrogen Oxidation Reaction. *J. Am. Chem. Soc.* **2024**, *146* (24), 16619–16629. https://doi.org/10.1021/jacs.4c03622.

[5] Han, L.; Ou, P.; Liu, W.; Wang, X.; Wang, H.-T.; Zhang, R.; Pao, C.-W.; Liu, X.; Pong, W.-F.; Song, J.; Zhuang, Z.; Mirkin, M. V.; Luo, J.; Xin, H. L. Design of Ru-Ni Diatomic Sites for Efficient Alkaline Hydrogen Oxidation. *Science Advances* **2022**, *8* (22), eabm3779. https://doi.org/10.1126/sciadv.abm3779.

[6] Xiao, W.; Lei, W.; Wang, J.; Gao, G.; Zhao, T.; Cordeiro, M. A. L.; Lin, R.; Gong, M.; Guo, X.; Stavitski, E.; Xin, H. L.; Zhu, Y.; Wang, D. Tuning the Electrocatalytic Activity of Pt by Structurally Ordered PdFe/C for the Hydrogen Oxidation Reaction in Alkaline Media. *J. Mater. Chem. A* **2018**, *6* (24), 11346–11352. https://doi.org/10.1039/C8TA03250E.

[7] Zhao, T.; Hu, Y.; Gong, M.; Lin, R.; Deng, S.; Lu, Y.; Liu, X.; Chen, Y.; Shen, T.; Hu, Y.; Han, L.; Xin, H.; Chen, S.; Wang, D. Electronic Structure and Oxophilicity Optimization of Mono-Layer Pt for Efficient Electrocatalysis. *Nano Energy* **2020**, *74*, 104877. https://doi.org/10.1016/j.nanoen.2020.104877.

[8] Shen, D.; Sun, F.; Liang, Z.; Mei, B.; Xie, Y.; Wang, Y.; Wang, L.; Fu, H. Oxygen Spillover on Supported Pt-Cluster for Anti-CO-Poisoning Hydrogen Oxidation. *Nat Commun* **2025**, *16* (1), 3883. https://doi.org/10.1038/s41467-025-58735-0.

[9] Zhao, Y.; Wu, D.; Luo, W. Correlating Alkaline Hydrogen Electrocatalysis and Hydroxide Binding Energies on Mo-Modified Ru Catalysts. *ACS Sustainable Chem. Eng.* **2022**, *10* (4), 1616–1623. https://doi.org/10.1021/acssuschemeng.1c07306.

[10] Xue, Y.; Shi, L.; Liu, X.; Fang, J.; Wang, X.; Setzler, B. P.; Zhu, W.; Yan, Y.; Zhuang, Z. A Highly-Active, Stable and Low-Cost Platinum-Free Anode Catalyst Based on RuNi for Hydroxide Exchange Membrane Fuel Cells. *Nat Commun* **2020**, *11* (1), 5651. https://doi.org/10.1038/s41467-020-19413-5.

[11] Cui, Z.; Ren, Z.; Ma, C.; Chen, B.; Chen, G.; Lu, R.; Zhu, W.; Gan, T.; Wang, Z.; Zhuang, Z.; Han, Y. Dilute RuCo Alloy Synergizing Single Ru and Co Atoms as Efficient and CO-Resistant Anode Catalyst for Anion Exchange Membrane Fuel Cells. *Angewandte Chemie International Edition* **2024**, *63* (28), e202404761. https://doi.org/10.1002/anie.202404761.

[12] Wu, J.; Gao, X.; Liu, G.; Qiu, X.; Xia, Q.; Wang, X.; Zhu, W.; He, T.; Zhou, Y.; Feng, K.; Wang, J.; Huang, H.; Liu, Y.; Shao, M.; Kang, Z.; Zhang, X. Immobilizing Ordered Oxophilic Indium Sites on Platinum Enabling Efficient Hydrogen Oxidation in Alkaline Electrolyte. *J. Am. Chem. Soc.* **2024**, *146* (29), 20323–20332. https://doi.org/10.1021/jacs.4c05844.

[13] Wang, P.; Yang, Y.; Zheng, W.; Cheng, Z.; Wang, C.; Chen, S.; Wang, D.; Yang, J.; Shi, H.; Meng, P.; Wang, P.; Tong, H.; Chen, J.; Chen, Q. V–O Species-Doped Carbon Frameworks Loaded with Ru Nanoparticles as Highly Efficient and CO-Tolerant Catalysts for Alkaline Hydrogen Oxidation. *J. Am. Chem. Soc.* **2023**, *145* (50), 27867–27876. https://doi.org/10.1021/jacs.3c11734.

[14] Cai, B.; Shen, D.; Xie, Y.; Yan, H.; Wang, Y.; Chen, X.; Wang, L.; Fu, H. Unlocking Superior Hydrogen Oxidation and CO Poisoning Resistance on Pt Enabled by Tungsten Nitride-Mediated Electronic Modulation. *J. Am. Chem. Soc.* **2024**, *146* (48), 33193–33203. https://doi.org/10.1021/jacs.4c12720.
